# Structures of the Human Poly (ADP-Ribose) Glycohydrolase Catalytic Domain Confirm Catalytic Mechanism and Explain Inhibition by ADP-HPD Derivatives

**DOI:** 10.1371/journal.pone.0050889

**Published:** 2012-12-10

**Authors:** Julie A. Tucker, Neil Bennett, Claire Brassington, Stephen T. Durant, Giles Hassall, Geoff Holdgate, Mark McAlister, J. Willem M. Nissink, Caroline Truman, Martin Watson

**Affiliations:** Innovative Medicines, AstraZeneca UK Ltd., Macclesfield, Cheshire, United Kingdom; Medical School of Hannover, United States of America

## Abstract

Poly(ADP-ribose) glycohydrolase (PARG) is the only enzyme known to catalyse hydrolysis of the *O*-glycosidic linkages of ADP-ribose polymers, thereby reversing the effects of poly(ADP-ribose) polymerases. PARG deficiency leads to cell death whilst PARG depletion causes sensitisation to certain DNA damaging agents, implicating PARG as a potential therapeutic target in several disease areas. Efforts to develop small molecule inhibitors of PARG activity have until recently been hampered by a lack of structural information on PARG. We have used a combination of bio-informatic and experimental approaches to engineer a crystallisable, catalytically active fragment of human PARG (hPARG). Here, we present high-resolution structures of the catalytic domain of hPARG in unliganded form and in complex with three inhibitors: ADP-ribose (ADPR), adenosine 5′-diphosphate (hydroxymethyl)pyrrolidinediol (ADP-HPD) and 8-*n*-octyl-amino-ADP-HPD. Our structures confirm conservation of overall fold amongst mammalian PARG glycohydrolase domains, whilst revealing additional flexible regions in the catalytic site. These new structures rationalise a body of published mutational data and the reported structure-activity relationship for ADP-HPD based PARG inhibitors. In addition, we have developed and used biochemical, isothermal titration calorimetry and surface plasmon resonance assays to characterise the binding of inhibitors to our PARG protein, thus providing a starting point for the design of new inhibitors.

## Introduction

Single-strand breaks (SSBs) are the most frequent type of DNA lesion occurring in prokaryotic and eukaryotic cells. They commonly arise from direct attack of deoxyribose by intracellular reactive metabolites, as abortive intermediates of topoisomerase 1 activity, or as intermediates occurring as a result of base excision repair (BER) acting to resolve lesions induced by genotoxins such as DNA alkylating and methylating agents [1]. One of the earliest responses to SSBs is poly(ADP-ribose) (PAR) synthesis by the first-discovered member of the Poly(ADP-ribose) polymerase (PARP) family, PARP-1. PARP-1 rapidly binds to and is activated by DNA single- and double-strand breaks, resulting in covalent modification of itself and other target proteins with long chains of PAR. PARP-1 utilizes NAD^+^ in the mono-ADP-ribosylation of a PAR acceptor protein, typically on a glutamic acid residue. Elongation and/or branching of the chain then occurs between each ribose. PAR polymers average one branch every 20–50 ADP-ribose (ADPR) units. Physiologically, this polymerisation triggers local chromatin relaxation and recruitment of DNA repair factors. The presence of high levels of PAR in cells is, however, transient because the polymer is rapidly degraded by PAR glycohydrolase (PARG), the only enzyme known to catabolise PAR following DNA damage [2].

Human PARG (hPARG) exists in several alternatively spliced isoforms: a 110-kDa nuclear form, at least two cytoplasmic isoforms of 99 and 103 kDa, and two mitochondrial isoforms of 60 and 55 kDa, all arising from the same transcript [3]–[5]. PARG hydrolyzes the *O*-glycosidic linkages of PAR, liberating ADPR monomers and shorter PAR chains [6]. Bovine and human PARG have been reported to exhibit both endo- (debranching) and exo-glycosidic activity [7], [8], initially cleaving large branches from complex PAR polymers and then removing terminal ADPR units. Mono(ADPR) hydrolase or mono(ADPR) protein lyase activity then removes the final ADPR unit from the PAR acceptor protein (reviewed in [9]). The physiological roles of PARG activity have been extensively investigated. PARG interacts with XRCC1, the central scaffold protein key to BER and is thought to disassemble XRCC1 complexes after a repair reaction has been completed, perhaps in readiness for reassembly at other sites. It has also been suggested that the degradation of PAR by PARG supplies ATP at DNA damage loci to fuel processes such as the DNA ligase III-mediated DNA ligation step in BER [2], [10].

Depletion of PARG from human cells results in a dramatic sensitisation to a variety of DNA damaging agents and severely retarded rates of SSB repair (SSBR), to levels similar to those achieved by PARP-1 depletion [2]. The response of PARG-depleted cells to oxidative damage is less clear as a protective effect of siRNA-mediated PARG depletion to high concentrations of H_2_O_2_ was reported [11]. PARP-1 and PARG co-depletion has been shown not to slow the rate of SSBR any more effectively than depletion of PARP-1 or PARG alone, suggesting that PARP-1 and PARG act in concert to accelerate SSBR. Irradiated PARG-deficient cells have been shown to display centrosome amplification and accumulate aberrant mitotic structures leading to polyploidy or cell death by mitotic catastrophe [12]. Furthermore, shRNA directed against all PARG isoforms in mammalian cells results in reduced repair of single and double strand breaks and oxidised bases. Complete knockdown of all PARG isoforms in mice is embryonic lethal. Hypomorphic mutants are viable but exhibit sensitivity to alkylating agents and gamma irradiation [13]–[15]. Lastly, consistent with PARG involvement in SSBR, it was reported recently [16] that depletion of PARG activity, using either siRNA or the reported PARG inhibitor Gallotannin, in tumour cells deficient in the homologous recombination repair pathway resulted in selective sensitisation. This supports the application of a PARG inhibitor to exploit synthetic lethal-mediated killing of pathway defective tumour cells. Taken together, these effects suggest that PARG inhibition represents a potentially attractive anti-cancer therapeutic strategy. Furthermore, applications towards other pathologies have been suggested owing to reports of PARG depletion leading to pro-survival effects upon oxidative stress and being protective against renal or splanchnic/reperfusion injury in mice [17], [18].

With the exception of the ADPR analogue and transition-state mimetic adenosine 5′-diphosphate (hydroxymethyl)pyrrolidinediol (ADP-HPD) and a series of recently reported rhodanine analogues [19], both of which lack cell permeability, none of the currently available PARG inhibitors show sufficient potency or specificity to allow testing in a cellular context (reviewed in [19]–[21]). Development of potent and specific small molecule PARG inhibitors has until recently been hindered by lack of both robust high throughput screening methods and an hPARG crystal structure [22]. Recent work from three independent groups has illuminated the field by provision of the structures of PARG catalytic domains from bacteria [23], protozoa [24], rat [25] and mouse (**PDB ID: 4FC2**). These structures reveal unexpected similarity between the catalytic core of PARG and the ADPR binding macro-domains [26]. The conserved ADPR binding core is flanked by additional sub-domains which are specific to PARG and vary across kingdoms. PARG specific motifs within the macro-domain itself harbour the catalytic residues responsible for glycohydrolase activity. Differences in the disposition of residues around the ADPR binding site resulting from sequence differences between bacterial and eukaryotic PARG have been proposed to account for the reported manifestation of both endo- and exo-glycohydrolase activities by mammalian PARG [24], [25]. Structures of complexes with ADPR [23], [24] and ADP-HPD [23]–[25] confirm the ADPR binding site and reveal the basis for competitive inhibition by ADP-HPD. However, none of the reported crystal systems provide sufficient information to serve as a basis for a structure-based drug-design programme against hPARG. Differences in active site architecture between bacterial, protozoal and mammalian PARG limit the usefulness of the *Thermonospora curvata* (Tc) and *Tetrahymena thermophila* (Tt) PARG crystal systems. Access to the active site of murine PARG (mPARG) is hindered as a result of crystal contacts. Finally, although the structure of unliganded rat PARG (rPARG) is of good resolution, the ADP-HPD complex structure is too low resolution to adequately resolve the bound water structure and side-chain conformations in the inhibitor binding site, both of which will be important for structure-guided drug design.

We therefore sought to determine structures of the catalytic domain of hPARG both alone and in complex with the known inhibitors, ADPR, ADP-HPD and 8-*n*-octylamino-ADP-HPD (OA-ADP-HPD) and to develop biochemical and biophysical assays to characterise the binding of hPARG to these inhibitors in order to enhance our understanding of the PARG catalytic mechanism and provide a validated system to support a structure guided drug design programme.

## Results and Discussion

### Designing a construct of the hPARG catalytic domain suitable for structural studies

Production of a crystallisable fragment of the hPARG catalytic domain proved challenging; we have had to use an array of bio-informatic and experimental approaches to engineer a suitable construct. At the time this work was carried out, no precedent for a PARG structure was available. Naturally occurring, functionally active C-terminal fragments of human, murine and bovine PARG had been characterised, suggesting that the catalytic activity of PARG was wholly located within this conserved C-terminal region [4], [27], [28]. Recombinant expression of bovine, rat and human PARG in *Escherichia coli* had also been described [6], [27], [29]. In order to define a minimal catalytic domain construct suitable for structural studies, we created a consensus disorder prediction based on the results of the automated disorder prediction software servers RONN [30], DisEMBL [31] and PrDOS [32] for the hPARG sequence (**UNIProt ID: Q86W56**). We combined this with a secondary structure prediction (PSIpred [33]), hydropathy plots (Vector-NTI, Invitrogen) and information on splice variants, intron-exon boundaries [3], [4], [34] and areas of conservation derived from multiple sequence alignments using ClustalW [35]. Finally, we used the surface entropy prediction software server, SERp [36], to identify two potentially surface exposed patches of lysine, glutamate and glutamine residues. Patch one comprises residues Lys616, Gln617 and Lys618, whilst patch 2 comprises residues Glu688, Lys689 and Lys690.

**Figure 1:**
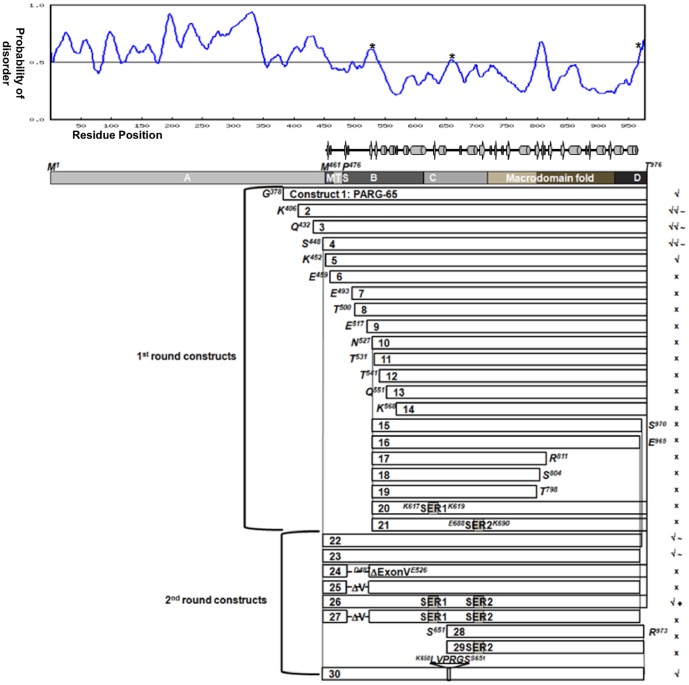
hPARG construct design. The 29 PARG fragments synthesized and tested for soluble expression, PARG activity and crystallisation are shown in relation to full-length hPARG(1–976) (hPARG). A representative disorder prediction (RONN, [30]) and a schematic of the secondary structure for hPARG26 are shown above the hPARG domain diagram (domain boundaries based on reference [5]). Sites determined as sensitive to trypsin in limited proteolysis experiments are indicated above the disorder prediction as *. Experimental outcomes are indicated to the right of each construct thus: × no soluble expression, √ low level soluble expression, √√ high level soluble expression, ∼ no crystals observed and ♦ crystals obtained. SER1 = surface entropy reduction patch 1 (K616A, Q617A, K618A). SER2 = surface entropy reduction patch 2 (E688A, K689A, K690A). MTS = putative mitochondrial targeting signal.

In all, we designed 21 constructs (Figure 1). A first set (constructs 1 to 14 in Figure 1) combined 14 N-termini, spanning a region highlighted by the disorder prediction as likely to mark the start of a more ordered region, with the natural C-terminus (Thr976). A second set (constructs 15 to 19 in Figure 1) tested an additional 5 C-termini against a single truncated N-terminus (Asn527) chosen on the basis of the disorder prediction. A final pair of constructs (constructs 20 and 21 in Figure 1) incorporated alanine mutations at the two predicted surface-entropy patches into our preferred construct (Asn527-Thr976, construct 10 in Figure 1). All of these constructs were generated with an N-terminal, cleavable, 6His affinity tag and tested for soluble expression in *E. coli* at small scale. Of the 21 constructs, only those 5 with the longest N-termini (constructs 1 to 5) gave soluble expression, and only 3 of these (constructs 2, 3 and 4) could be purified in sufficient amounts to set up crystallisation trials (Figure 1). None of these constructs yielded crystals, despite extensive screening in the presence and absence of ligands (ADPR, ADP-HPD).

**Figure 2:**
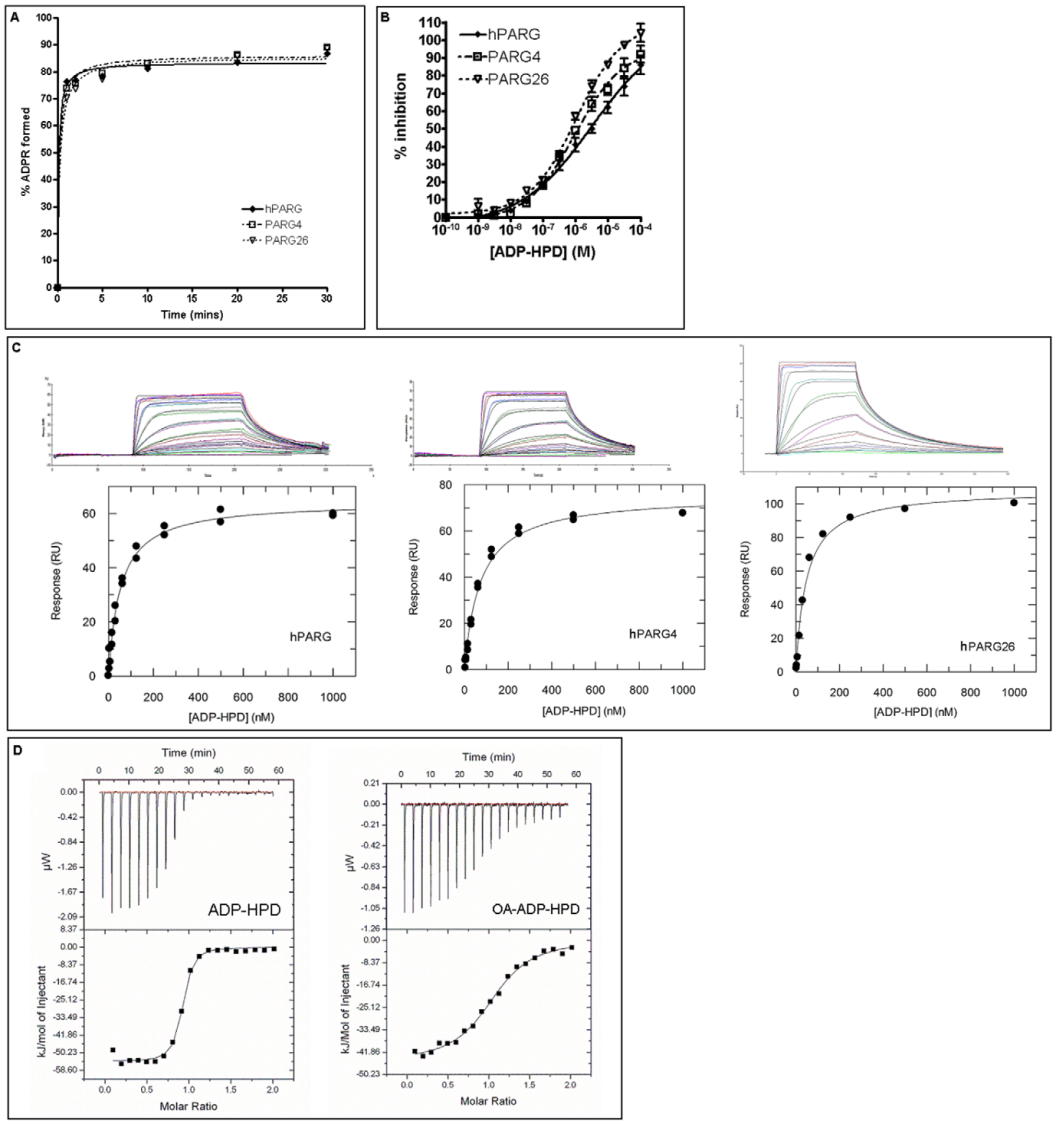
hPARG catalytic domain constructs show equivalent *in vitro* enzymatic activity and ADP-HPD binding properties as the full-length enzyme. (a) Time-course of PAR-PARP1 hydrolysis by recombinant PARG as measured in a homogeneous time-resolved fluorescence (HTRF) assay. Data points are the mean of three measurements carried out on separate occasions. (b) Inhibition of PAR-PARP1 hydrolysis by ADP-HPD. Data points are the mean of all measurements from three separate experiments, each run in triplicate ± Standard Error. Percent inhibition was calculated with respect to “No Enzyme” and “No Inhibitor” controls. (c) Representative binding sensorgrams and steady state fits for ADP-HPD binding to immobilised hPARG, hPARG4 and hPARG26 monitored by SPR. (d) Representative binding isotherms showing binding of ADP-HPD and OA-ADP-HPD to hPARG4 monitored by ITC.

We chose to further characterise the shortest construct giving viable expression levels. Construct 4, hPARG(448–976), hereafter referred to as hPARG4, showed equivalent enzyme activity to full-length recombinant hPARG (hPARG(1–976)-6His, hereafter referred to as hPARG) in a PAR-PARP1 hydrolysis assay (Figure 2a). hPARG and hPARG4 showed similar IC_50_ values for inhibition by ADP-HPD (Table 1 and Figure 2b), and these were in good agreement with literature values for bovine PARG (bPARG; 0.33 µM for purified full-length protein and 1–1.4 µM for recombinant catalytic domain [37]). hPARG4 was found to be suitable for ligand-observed nuclear magnetic resonance (NMR), isothermal titration calorimetry (ITC) and Surface Plasmon Resonance (SPR) immobilisation experiments, giving high-quality SPR binding curves and ITC isotherms (Figures 2c and 2d). We determined a K_D_ for ADP-HPD equivalent to that of the full-length protein within experimental error (Table 1 and Figures 2c, 2d and S2) and in reasonable agreement with literature values for bPARG (50–80 nM [38]).

**Table 1:** ADP-HPD inhibition and binding data for human PARG constructs.

Construct	Compound	IC50 (µM)	K_D_ (nM) (SPR)	K_D_ (nM) (ITC)	ΔH (kJ/mol)	ΔS (J/mol/K)	ΔG (kJ/mol)
hPARG	ADP-HPD	3.1±0.2 (n = 3)	44.8±2.9 (n = 2)	416±89.9 (n = 1)	−30.8±0.04	+18.8±1.7	−36.4±0.5
hPARG	OA-ADP-HPD	16.3±1.1 (n = 6)	n.d.	n.d.	n.d.	n.d.	n.d.
hPARG4	ADP-HPD	0.97±0.24 (n = 3)	64.3±2.6 (n = 3)	132±14 (n = 3)	−56.1±0.4	−56.1±0.5	−39.4±0.3
hPARG4	OA-ADP-HPD	n.d.	n.d.	800±100 (n = 2)	−50.6±1.4	−52.9±4.7	−34.9±0.3
hPARG26	ADP-HPD	1.10±0.22 (n = 3)	50.0±6.4 (n = 1)	n.d.	n.d.	n.d.	n.d.

Replicates are indicated in parentheses. IC_50_ values are arithmetic means ± standard errors. Values quoted for the SPR and ITC data are arithmetic means ± absolute errors calculated by propagation of errors. SPR binding constants were derived from steady state fits. n.d. = not determined.

Based on the knowledge gained from our experience with the initial set of 21 constructs regarding stable N- and C-termini, combined with limited proteolysis experiments, and additional literature information, we designed a second set of constructs (Figure 1, constructs 22–29). We kept the N-terminus at Ser448 and sampled 3 C-termini, six surface-entropy reduction mutations, an internal deletion (corresponding to a natural splice variant, Δ488–526 [4]), and an inserted thrombin cleavage site centred on residue 651 (identified by limited proteolysis). The longer constructs showed soluble expression and could be purified (Figure 1, constructs 22, 26 and 29). Interestingly, although thrombin cleavage of construct 29 was successful, the two resulting fragments co-purified and could not be separated under native conditions. In combination with the lack of soluble expression for constructs 27 and 28, this suggested that the stable C-terminal fragment identified by limited proteolysis did not constitute a discrete domain.

The only construct to give crystals was construct 26, hPARG (448–976 [K616A, Q617A, K618A, E688A, K689A & K690A]), hereafter referred to as hPARG26, which contained all six surface-entropy reduction mutations. The six mutations required for crystallisation did not affect the activity of hPARG26 compared to hPARG4 in our PAR-PARP1 depolymerisation assay, nor did they alter the IC_50_ for ADP-HPD (Table 1 and Figures 2a and 2b), confirming the integrity of the catalytic and PAR binding sites.

### Crystallisation & Structure Solution

Sparse matrix crystallisation trials yielded crystals of unliganded hPARG26, which showed diffraction to beyond 2 Å on a rotating anode X-ray generator. At the time this work was carried out, no suitable model was available for molecular replacement. Efforts to solve the structure by isomorphous replacement using a variety of heavy atoms or sulphur anomalous dispersion techniques were unsuccessful. We therefore produced Selenomethionine (SeMet)-labelled protein and crystals to enable structure determination by the multiple wavelength anomalous dispersion (MAD) method. SeMets were located and the structure solved using data from a 4-wavelength MAD experiment carried out at the ESRF (full details are provided in the Materials and Methods and Table 2). SeMet-labelled hPARG26 crystallises in a monoclinic space group whilst unlabelled protein crystallises in an orthorhombic space group. A partially refined model built into the monoclinic SeMet hPARG26 data at 1.83 Å was used to solve the structure of the ligand-free orthorhombic unlabelled hPARG26 at 1.75 Å. There is one copy of hPARG26 in the crystal asymmetric unit in both crystal forms. The monoclinic and orthorhombic unliganded hPARG26 structures are essentially identical (r.m.s. deviation 0.38 Å over all Cα atoms), and further discussion focuses on the higher resolution orthorhombic structure. Co-ordinates have been deposited with the PDB under **PDB ID 4B1G** and **PDB ID 4A0D**.

**Table 2:** Crystallographic statistics for structures of monoclinic hPARG catalytic domain (SeMet-hPARG26).

Structure	Monoclinic SeMet hPARG26			
Crystal	**1**			**2**
PDB code	**n/a**			**PDB ID:4B1G**
X-ray source	ESRF, ID23-EH1			ESRF, ID23-EH1
Wavelength (Å)	0.9788 (peak)	0.9795 (inflection)	0.9754 (remote)	0.979 (inflection)
Resolution (Å) (outer shell)	88.5–1.85^a^ (1.95–1.85)	88.6–1.89^a^ (2.0–1.89)	88.6–2.13^a^ (2.25–2.13)	1.83 (1.93–1.83)
Observations	302879	273450	216888	250308
Unique reflections	43181	40767	29277	39497
Completeness (%)(outer shell)	96.9 (81.6)	98.4 (89.5)	100 (100)	86.3 (47.9)
Rmeas (outer shell)	0.063 (0.46)	0.077 (0.468)	0.072 (0.497)	0.106 (0.375)
mean I/σ(I) (outer shell)	18.2 (2.8)	15.5 (1.9)	17.8 (3.9)	12.4 (2)
Protein atoms	3479			4057
Water atoms	0			294
Other heteroatoms	80			99
Crystallographic R (%) (outer shell)	25.6 (44.1)^b^			18.5 (29.7)
Rfree (%) (outer shell)	27.1 (44.9)			21.8 (34.8)
Mean B (Å^2^) (protein; water; ligand; other heteroatoms)	21.6; null; null; 25.1			19.4; 24.1; null; 26.1
RMS bond length (Å)	0.012			0.015
RMS angle (°)	1.49			1.44

a Effective resolution limited to 2.3 Å by ice rings.

b Model partially refined against peak data.

### Structure of the hPARG catalytic domain

**Figure 3:**
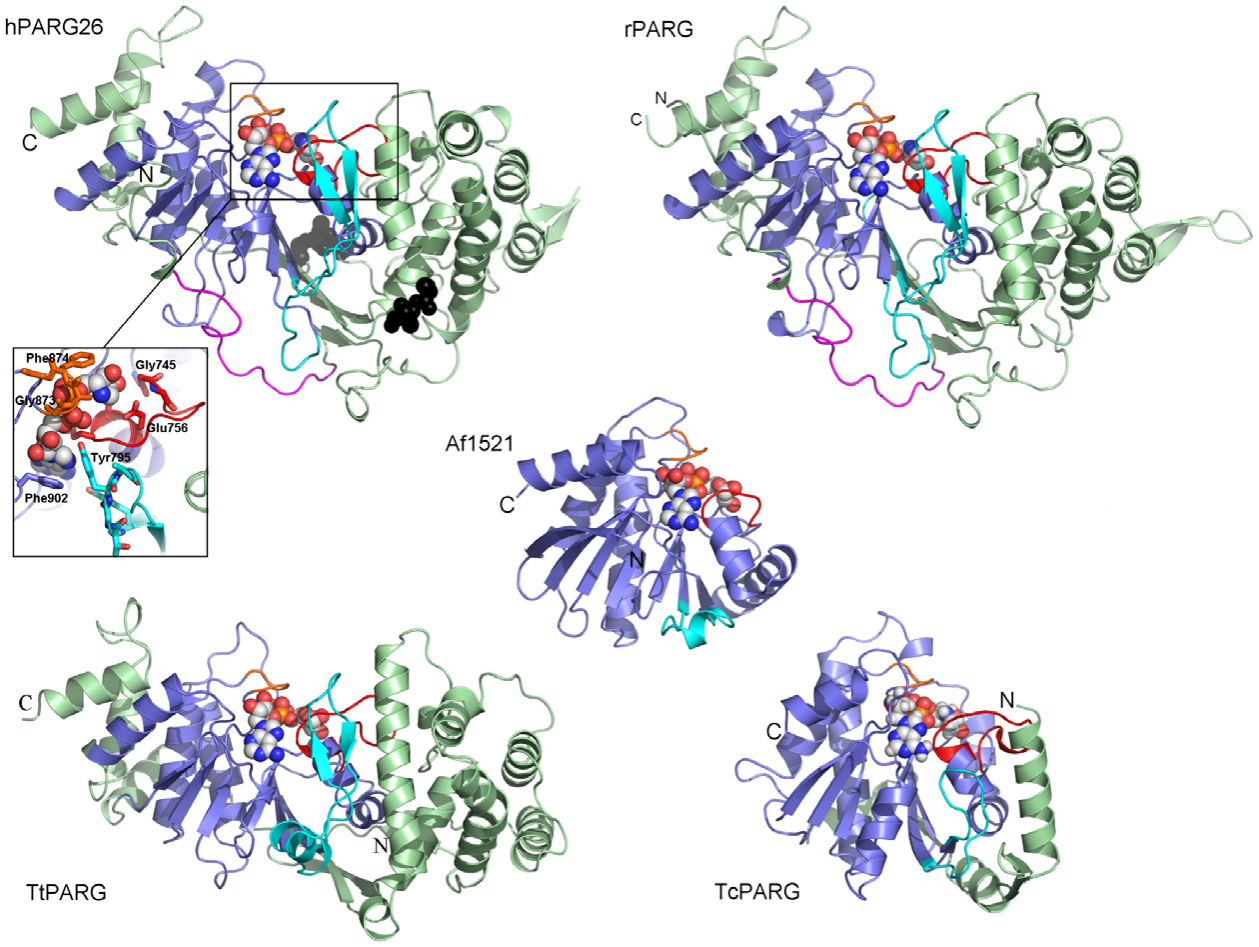
Comparison of hPARG catalytic domain with representative structures of mammalian, protozoal and bacterial PARG catalytic domains and an ADPR-binding macrodomain. Clockwise from top left: hPARG26 in complex with ADP-HPD (**PDB ID: 4B1J**); the Cα atoms for the six SER point mutations in hPARG26 are shown as black spheres. rPARG in complex with ADP-HPD (**PDB ID: 3UEL**). TcPARG in complex with ADP-HPD (**PDB ID: 3SII**). TtPARG in complex with ADPR (**PDB ID: 4EPP**). Inset: close up of the hPARG26 active site; rotated 90° towards the viewer with respect to main panel; selected side-chains are drawn in stick representation and labelled. Centre: Af1521 in complex with ADPR (**PDB ID: 2BFQ**). The conserved macrodomain fold is coloured blue, with N- and C-terminal extensions in pale-green. N- and C-termini are labelled. The PARG-specific GGG-X_6–8_-QEE motif is highlighted in red, as is the corresponding Gly-rich loop in Af1521. The phosphate binding loop (G^873^AFG in hPARG) is highlighted in orange. The “Tyr-clasp” [25] and equivalent regions in TtPARG, TcPARG and Af1521 are highlighted in cyan. The MTS in hPARG26 and rPARG is highlighted in magenta. Bound ADP-HPD and ADPR are drawn as spheres with carbon in grey. Colour scheme adapted from [26]. Figures were prepared using PyMol (Schrödinger LLC).

The structure of hPARG26 comprises a twisted, mixed, 10-stranded β-sheet core flanked by two predominantly α-helical sub-domains arranged so as to form a central cleft above the β-sheet (Figure 3). The extents of visible electron density agree well with the consensus disorder prediction used in the construct design, whilst the observed sensitivity of certain regions to trypsin cleavage correlates with their location in surface exposed, partially-ordered loops (Gly527 and Ser651) or with the C-terminal extent of the electron density (Glu965, Figures 1 and S1).

Comparison of the hPARG26 structure with other structures in the Protein Data Bank (PDB) highlighted structural similarity between a highly conserved stretch of ∼200 amino acids in the C-terminal portion of the PARG catalytic domain and an ADP-ribose binding macro domain from *Archaeoglobus fulgidus*, Af1521 [39] (highlighted as macrodomain within sub-domains C and D in Figure 1, blue ribbon in Figure 3). This conservation of fold despite significant sequence dissimilarity (<12% sequence identity) has been confirmed in subsequent structures of a bacterial PARG [23], a protozoal PARG [24] and two mammalian PARGs ([25] and **PDB ID: 4FC2**) (Figure 3). These latter structures confirm the conservation of overall fold amongst mammalian PARG catalytic domains as expected given their high level of sequence conservation (>90% sequence identity). Comparison of mammalian with bacterial and protozoal PARG, on the other hand, serves to highlight the diversity in accessory regions around the conserved ADPR binding macrodomain core (pale-green ribbons in Figure 3).

As noted by Kim and co-workers [25], the mammalian PARG catalytic domain is considerably extended at both the N- and C-termini compared to the bacterial PARG [23] and the Af1521 macrodomain [39]. The N-terminal extension comprises ∼270 residues. The N-terminal residues of this extension which contain the proposed mitochondrial targeting sequence [5] (MTS; residues 461 to 477, magenta ribbon in Figure 3) extend in a well-defined loop which wraps around the C-terminal extension, and then crosses the macro-domain core, contributing an additional short β-strand to the central β-sheet, before joining with the N-terminal helical sub-domain (residues 535 to 715, pale-green ribbon on right of hPARG26 in Figure 3) via a trypsin sensitive β-hairpin. Although largely lacking in secondary structure itself, the MTS loop is integral to the catalytic domain structure, shielding a highly hydrophobic patch (centred on Trp814), explaining why truncations beyond Lys452 resulted in insoluble expression. The C-terminal extension comprises ∼80 residues which fold into a three-helix motif that packs on the opposite face of the PARG catalytic core to the N-terminal helical extension (pale-green ribbon on left of hPARG26 in Figure 3).

**Figure 4:**
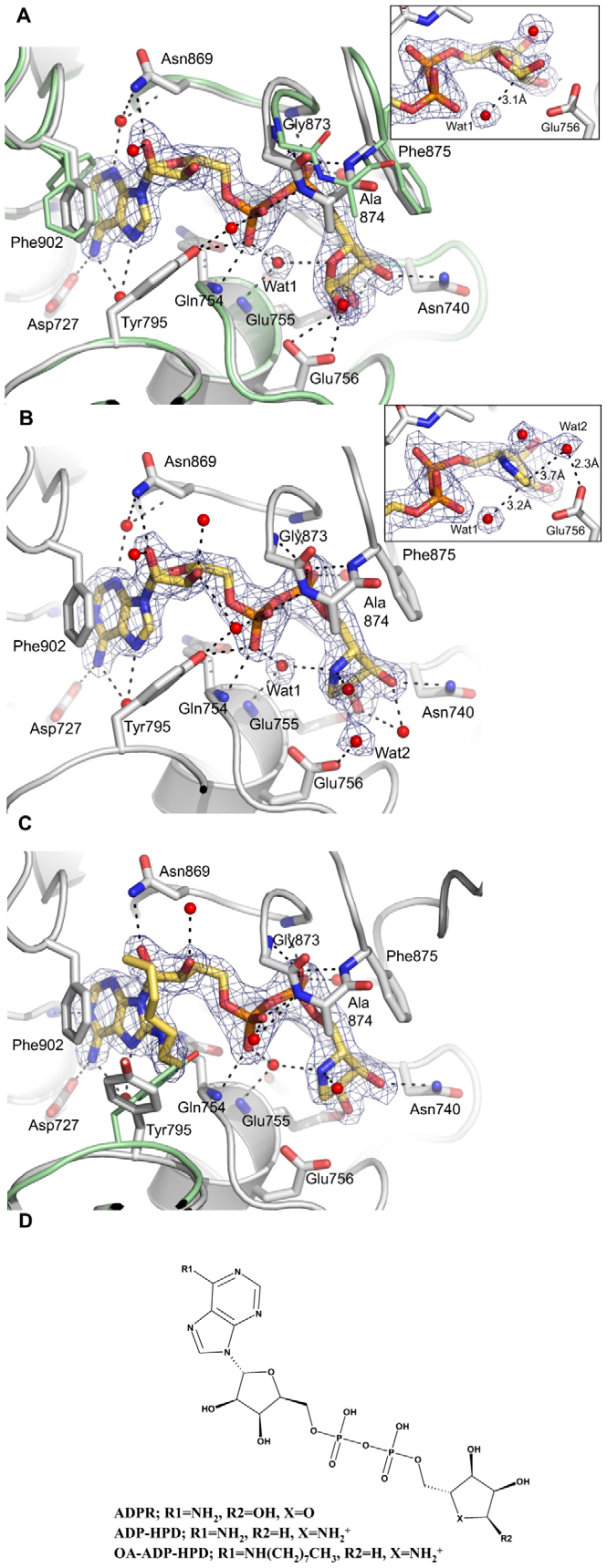
Binding of ADPR, ADP-HPD and OA-ADP-HPD is accompanied by conformational changes in the active site of human PARG. 2Fo-Fc omit maps for bound ligand and waters are shown in blue contoured at 1σ. Pictures prepared using PyMol (Schrödinger, LLC). (a) hPARG26 in complex with ADPR. Electron density clearly reveals binding of the α-anomer. A tightly bound water molecule (Wat1, also present in the unliganded structure), positioned 3.1 Å from the ribose” anomeric carbon, has been proposed to act as a nucleophile during hydrolysis [23], [25]. The ribose” 1″-OH PAR attachment site lies 2.6 Å from Oε2 of the putative catalytic acid/base, Glu756 (see inset). Overlay of ADPR-bound (grey) and unliganded (pale-green) structures highlights closure of the conserved G^873^AFG loop over the di-phosphate moiety, and rotation of the Phe902 side-chain out of the adenine pocket upon ADPR binding. (b) hPARG26 in complex with the transition-state mimetic, ADP-HPD. As noted in the ADPR complex, a water molecule (Wat1) lies close to the anomeric carbon below the plane of the HPD-ring. In the ADP-HPD complex, a second water molecule (Wat2) lies 3.7 Å from the anomeric carbon above the plane of the HPD-ring and within H-bond distance (<2.3 Å) of the Glu756 side-chain (see inset). Both of these waters are also present in the unliganded hPARG26 structure. Either could generate product (ADPR) by nucleophilic attack on the transition-state after cleavage of the scissile bond. (c) hPARG26 in complex with OA-ADP-HPD. Overlay of OA-ADP-HPD-bound (grey) and ADP-HPD-bound (pale-green) structures highlights rotation of the Tyr-795 side-chain to accommodate the 8-*n*-octylamino moiety. (d) 2D structure depiction of compounds used in this study.

The cleft above the central β-sheet in Figure 3 is lined by highly conserved residues implicated in ADPR binding and PAR hydrolysis (Figure 3, inset). One side of the cavity is flanked by the diphosphate binding loop (G^873^AFG, orange in Figure 3), whilst the other comprises the PARG specific GGG-X_6–8_-QEE motif (residues 744–756, red in Figure 3 [6]), placing the conserved catalytic residues (Q^755^EE) at one end of the cleft (away from the viewer in Figure 3, see inset). The cleft is further bounded by a third conserved motif, specific to eukaryotic PARG, with the sequence YTGYA [6] (residues 792–796, cyan in Figure 3). This sequence lies at the centre of a structural motif first identified in rPARG and termed the “Tyr-clasp” [25]. Complex structures with ADPR and ADP-HPD confirm the cleft as the primary ADPR binding site and catalytic centre (this work; **PDB ID:** **4B1H**, **PDB ID: 3UEL** [25] and **PDB ID: 4EPP** [24]) and reveal that, in the absence of ligand, the ADPR binding site is partially blocked by the side-chain of a conserved phenaylalanine (Phe902 in hPARG, pale-green in Figure 4a) (this work; **PDB ID:** **4A0D**, **PDB ID: 3UEK** [25] and **PDB ID: 4FC2**).

A number of sulphate ions and glycerol molecules derived from the crystallisation buffer were observed to bind to pockets on the surface of the N-terminal helical extension. A deep, water-filled pocket is located on the opposite face of the macro-domain core to the ADPR binding site. These pockets may represent areas for interaction with the polymeric PAR substrate, other regions of the PARG protein absent from this construct, and PARP1, which has been shown to interact with PARG [40] (Truman, C., unpublished data).

Consistent with their lack of effect on PARG catalytic activity and ADP-HPD binding, the two surface entropy mutation triplets are located on surface loops within the C-terminal extension and distal to the ADPR binding site (drawn as black spheres on hPARG26 in Figure 3). Comparison of the hPARG26 structure with rPARG and mPARG structures confirms the limited effect of these mutations on the local structure. Mutation of Glu688 to Ala results in loss of an H-bond to Arg684, which consequently re-orients to pick up alternative interactions with Ser592, Asp596 and Thr687. Mutation of Lys690 to Ala is accompanied by re-orientation of Glu628 to pick up alternative water-mediated interactions. Interestingly, in the rPARG structure Lys615 (equivalent to Lys619 in hPARG) participates in a crystal contact, such that the K619A mutation in hPARG26 has removed a potential contact. It would appear that the beneficial effect of the surface-entropy mutations on crystallisation propensity arises from an overall reduction in surface entropy [41], [42] and local changes in surface charge, rather than the formation of any new crystal contacts.

Crystals of hPARG26 were robust, tolerating soaking with a variety of ligands. We were thus able to generate high resolution structures of complexes with the reaction product, ADPR, and two known inhibitors, ADP-HPD [38] and OA-ADP-HPD [37] in order to increase our understanding of the mechanisms of PARG catalysis and inhibition. Detailed statistics for the data collection and model refinement and PDB accession codes are provided in Table 3.

**Table 3:** Crystallographic statistics for structures of orthorhombic hPARG catalytic domain (hPARG26).

Structure	Orthorhombic hPARG26	hPARG26-ADPR	hPARG26-ADPHPD	hPARG26-OA-ADP-HPD
PDB code	**PDB ID: 4A0D**	**PDB ID: 4B1H**	**PDB ID: 4B1J**	**PDB ID: 4B1I**
X-ray source	ESRF, ID29	Rigaku FRE	Rigaku FRE	Rigaku FRE
Wavelength (Å)	0.98	1.54	1.54	1.54
Resolution (Å) (outer shell)	1.75 (1.84–1.75)	2.0 (2.07–2.0)	2.08 (2.19–2.08)	2.14 (2.26–2.14)
Observations	57354	39298	32176	31210
Unique reflections	194594	241741	202691	195851
Completeness (%)(outer shell)	97.7 (97)	98.9 (90.8)	90.2 (52.7)	97.5 (84.4)
Rmeas (outer shell)	0.062 (0.434)	0.073 (0.396)	0.11 (0.487)	0.105 (0.473)
mean I/σ(I) (outer shell)	9 (2.0)	10.8 (2.5)	12.4 (2.8)	13.7 (2.9)
Protein atoms	4228	4130	4157	4135
Water atoms	411	378	529	489
Other heteroatoms	54	106	62	77
Crystallographic R (%) (outer shell)	16.6 (25.4)	20.5 (35.0)	16.8 (23.9)	15.9 (23.0)
Rfree (%) (outer shell)	19.6 (27.9)	24.1 (40.1)	21.5 (26.5)	20.7 (26.6)
Mean B (Å^2^) (protein; water; ligand; other heteroatoms)	26.6; 52.8; null; 36.4	21.3; 28.2; 35.7; 44.9	18.7; 27.9; 24.7; 29.1	35.9; 45.1; 37.1; 77.2
RMS bond length (Å)	0.014	0.015	0.014	0.014
RMS angle (°)	1.453	1.41	1.41	1.41

### Structure of the hPARG26 ADPR complex

**Figure 5:**
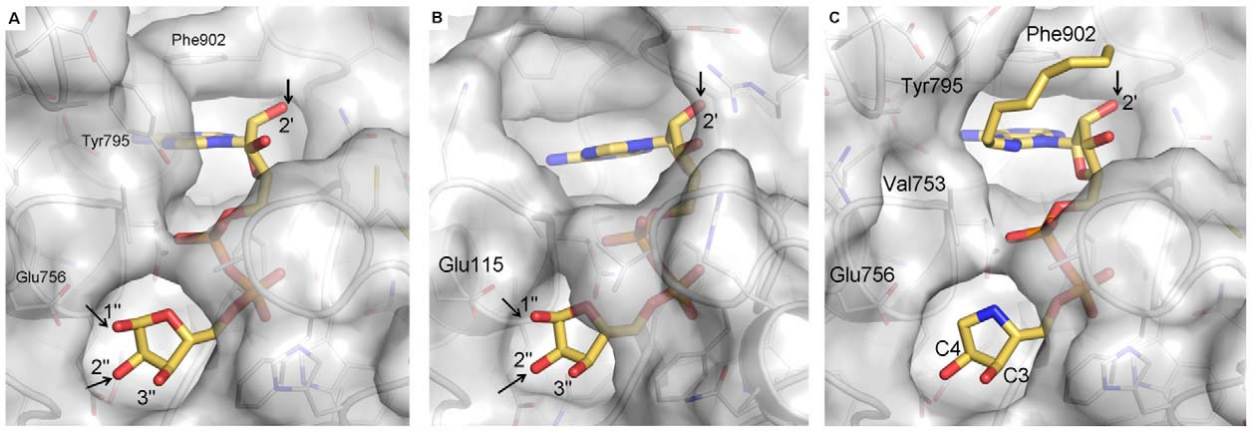
Differences in solvent accessibility of bound ADPR between hPARG (a) and TcPARG (b) may account for the reported endo- and exo-glycohydrolase activity of mammalian PARG and predict different SAR for ADP-HPD derived inhibitors (c). (a) hPARG26-ADPR structure with transparent surface drawn over the protein. ADPR is shown in stick representation with carbons in yellow. Selected protein residues are labeled. Ribose hydroxyls are labeled and potential PAR linkage points indicated with an arrow. Of these, the 1″ and 2′-OH are accessible. (b) as (a) for TcPARG (**PDB ID: 3SIG**). (c) as (a) for hPARG26-OA-ADP-HPD (**PDB ID: 4B1I**). HPD carbons C3 and C4 are labeled.

ADPR binds to the hPARG26 catalytic cleft in a similar manner to that observed in the TcPARG and TtPARG structures [23], [24] and other ADPR binding macrodomains ([39]; **PDB ID: 3V2B**; **PDB ID: 3Q71**). Binding of ADPR is accompanied by conformational changes in the vicinity of the binding pocket that effectively shield the bound ligand from solvent (Figures 4a and 5a); specifically closure of the phosphate binding loop, and concerted movement of the adjacent β12-α10 loop (H^828^FRR), rotation of the Phe902 side-chain out of the adenine pocket, and inward movement of the single-turn α7 helix and preceding loop (G^724^TIEENG). These rearrangements are more extensive than those observed in TcPARG, where the active site appears to be effectively pre-formed.

**Figure 6:**
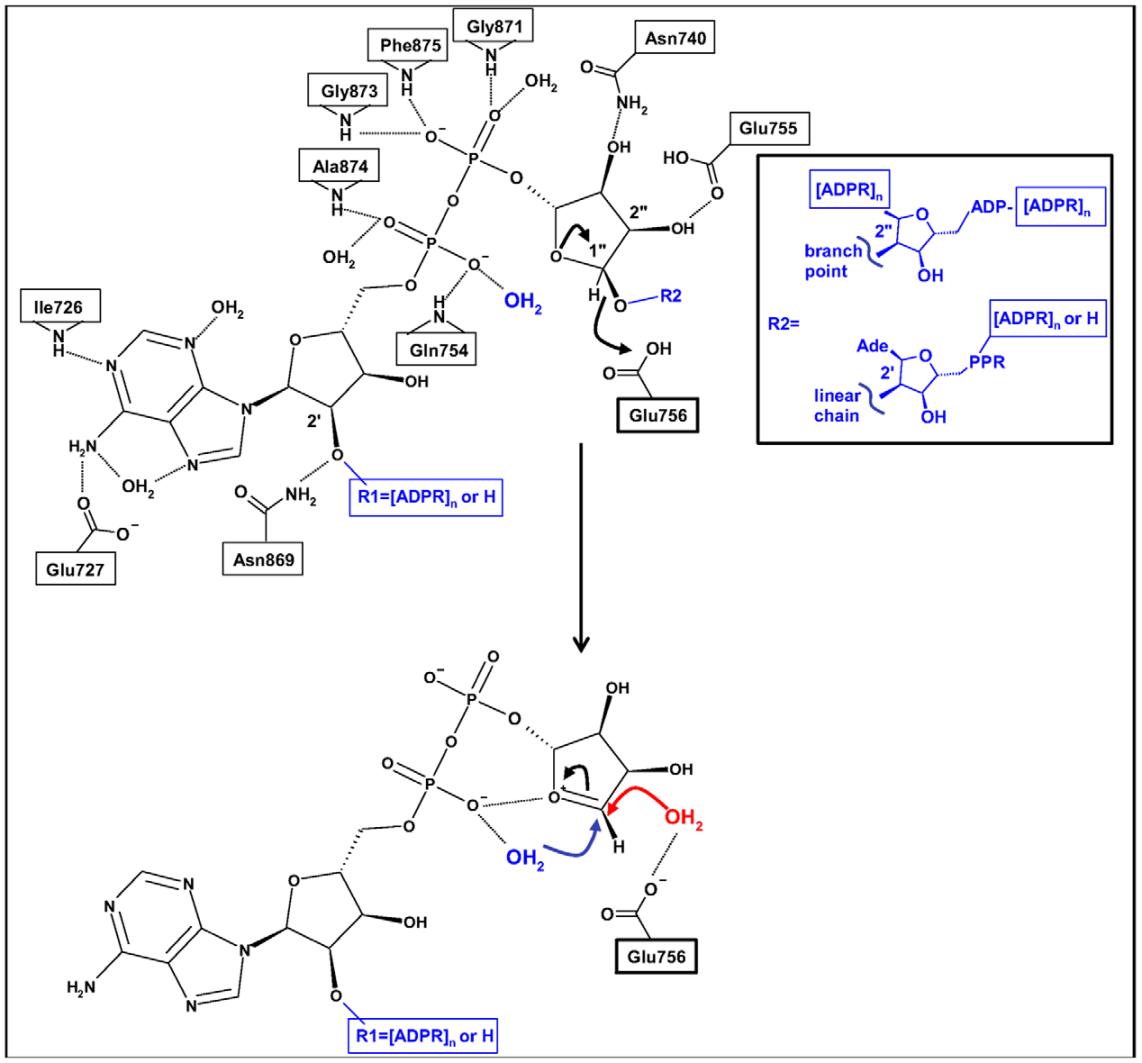
Schematic structure-based mechanism for the reported endo- and exo-glycohydrolase activities in hPARG. Selected interacting residues and water molecules are shown, with H-bonds drawn as dashed lines. The terminal ADPR unit (R1 = H) or possibly also an internal ADPR unit (R1 = PAR) within a linear (R2 = linear chain) or branched (R2 = branch point) PAR chain bind to the hPARG catalytic site. Glu756 acts as the catalytic acid/base to effect cleavage of the scissile ribose” 1″-O-R2 bond, releasing shorter, linear PAR and possibly also de-branched PAR. The oxocarbenium ion intermediate undergoes nucleophilic attack by one of two water molecules via an inverting (Wat1, blue) or retaining (Wat2, red) mechanism, to generate ADPR (R1 = H, exo-glycohydrolysis), or possibly also shorter PAR (R1 = PAR, endo-glycohydrolysis).

Comparison of unliganded and ADPR-bound hPARG26 structures shows that the adenine binding site is partially blocked by the side-chain of Phe902 in the absence of ligand (Figure 4a, Phe902 side-chain from unliganded hPARG26 highlighted in pale-green). Steric block of the adenine site was also observed in the unliganded structures of rPARG and mPARG ([25]; **PDB ID: 4FC2**). In hPARG26, the electron density for the Phe902 side-chain could best be modelled in two conformations, one of which is more similar to that observed in the unliganded rPARG and mPARG structures whilst the other is more similar to that observed in the ADPR complex. This suggests that Phe902 may be more flexible and easily displaced in hPARG than in mPARG and rPARG, where the electron density is consistent with a single side-chain rotamer. Upon ADPR binding, Phe902 rotates to stack against the adenine moiety, which is additionally held in place by a network of direct and water-mediated hydrogen bonds (H-bonds) (Figures 4a and 6). In particular, the 6-amino group donates an H-bond to the side-chain of Glu727, a residue conserved across eukaryotic PARGs, and a contact unique to eukaryotic PARG structures. N1 accepts a H-bond from the backbone NH of Ile726. N3 and N7 are involved in water-mediated H-bonds to the backbone of Gln754, Asn869 and Phe900, and side-chain atoms of Glu727 and Tyr792.

The ribose' moiety is secured by an H-bond between the 2′-OH and the side-chain amino of Asn869, a contact unique to the mammalian PARG structures (corresponding residue in TtPARG is Lys365). The ribose' 1′-OH is exposed to solvent.

The diphosphate moiety is securely anchored by H-bonds to the backbone NH of Gln754 from the conserved QEE motif, and residues in the phosphate binding loop, specifically Gly871, Gly873, Ala874 and Phe875 (Figures 4a and 6). The tip of this loop moves by ∼2 Å upon ADPR binding (Figure 4a, Cα trace for unliganded hPARG26 highlighted in pale-green). Phe875, at the tip of the phosphate binding loop, forms one face of the ribose” site. Closure of the phosphate binding loop is accompanied by movement in the adjacent β12-α10 loop (H^828^FRR). In the mPARG structure (**PDB ID: 4FC2**), the side-chain of Phe868 (equivalent to hPARG Phe875) occupies the phosphate and ribose” binding sites. Movement of the Phe868 side-chain is constrained by proximity to the side-chain of His821. His821 is prevented from adopting the rotamer observed in hPARG26 by the side-chain of Arg823, which is in turn held in place by contacts with the side-chains of Glu954 (H-bond) and Tyr950 (π-stack) from a symmetry-related molecule.

The terminal ribose” is located in the vicinity of the conserved acidic residues, Asp737, Glu755 and Glu756, such that the 1″-, 2″- and 3″-hydroxyl groups contact Glu756, Glu755 and Asn740 respectively (Figures 4a and 6). ADPR is bound as the α-anomer, consistent with the known stereochemistry of PAR linkages [43]–[45] (Figure 4a inset).

**Figure 7:**
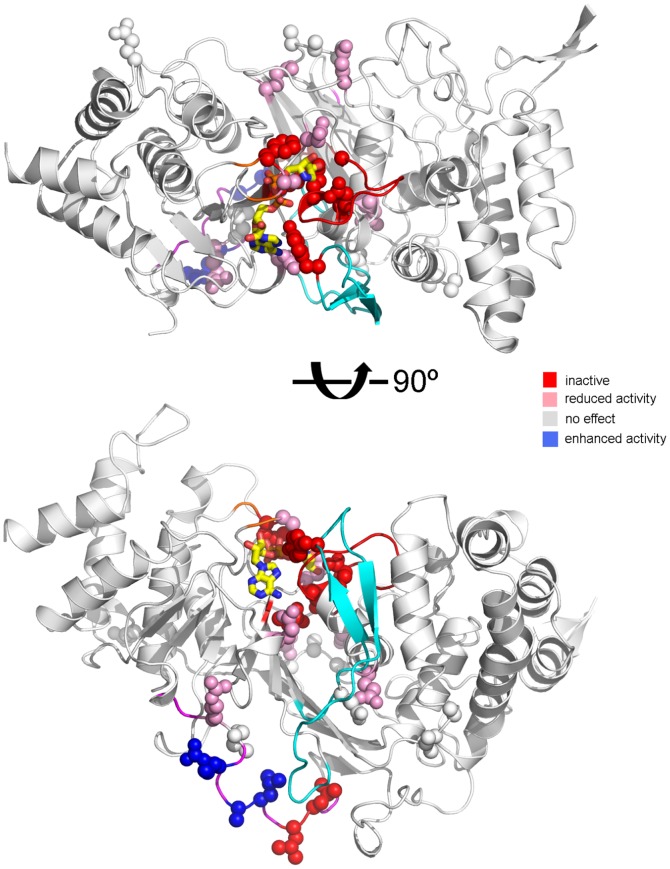
Mapping site-directed mutations onto the hPARG26 structure explains their effect on PARG activity. The hPARG26-ADP-HPD structure is shown in ribbon representation coloured to highlight important structural motifs as in Figure 3. Bound ADP-HPD is drawn as sticks with carbon atoms in yellow. Side-chains of Trp814 and Pro472 are drawn as sticks, with the H-bond between the Trp814 indole NH and Pro472 backbone CO shown as a dashed black line. Mutated residues are drawn as spheres and coloured according to their effect on PARG activity from red (activity abolished) through white (no effect) to blue (activity enhanced). See Table S1 for further details of individual mutations.

Patel and co-workers [6] proposed a catalytic rôle for three conserved acidic residues, Asp737, Glu755 and Glu756 (hPARG numbering), on the basis of site-directed mutagenesis experiments using bPARG (Figure 7 and Table S1). The hPARG26-ADPR structure reveals that Glu756 accepts an H-bond from the ribose” 1″-OH, the expected site of attachment for a second ADPR in a linear PAR chain, and thus likely constitutes the catalytic acid/base. Consistent with the bPARG mutational data, replacement of Glu756 with Asn would be predicted to abolish catalytic activity by removing the catalytic acid. Glu755 accepts an H-bond from the ribose” 2″-OH, contributing to stabilization and orientation of the ADPR conformation. Asp737 is buried, and appears to perform a structural role stabilizing the conformation of Glu755, whilst also potentially acting as part of a proton relay network to Glu756 [24]. Consistent with this latter hypothesis, the D738N and E756N mutations in bPARG (equivalent to D737N and E755N in hPARG) abolish catalytic activity without significant negative impact on the binding of 8-aminohexylaminoADP-HPD (8-AH-ADP-HPD) [6], suggesting that whilst the shape of the binding pocket is maintained in these mutants, the proton relay network is disrupted. Mutation of Glu114 to Ala in TcPARG (equivalent to E755A in hPARG) on the other hand resulted in both a significantly reduced binding affinity for ADPR and loss of catalytic activity [23]. Slade and co-workers [6] similarly demonstrated a loss of catalytic activity for Glu755Ala, Glu756Ala and Glu756Asn mutants of hPARG (see Table S1).

In contrast to the TcPARG-ADPR complex, in hPARG26 both ribose' 2′-OH and ribose” 1″-OH PAR attachment points are accessible to solvent (Figures 5a, 5b and 6). This results from a difference in sequence between bacterial and eukaryotic PARG (Figure S1) which significantly shortens the loop covering the adenine binding site from seven residues down to three. On the basis of inaccessibility of the ribose' 2′-OH in the TcPARG complex structure, TcPARG was suggested to be exclusively an exo-glycohydrolase, binding and acting on the PAR terminus. Mammalian PARG, however, is reported to possess both endo- and exo-glycohydrolase activities [7], [8], [46]–[48]. In agreement with the mechanism proposed on the basis of the rPARG-ADP-HPD complex structure [25], accessibility of 2′ and 1″ ribose hydroxyls to solvent in ADPR-bound hPARG26 might allow binding of ADPR moieties adjacent to branch points and within linear chains (Figures 5a and 6) thus accounting for the reported endo- and exo-glycohydrolase activities. Subsequent cleavage at the 1″-OH linkage releases shorter, de-branched polymers thus accounting for the previously reported endo-glycohydrolase activity. The third potential PAR attachment point, ribose” 2″-OH, is sterically hindered such that ADPR at a branch point could not be bound.

A small, hydrophobic cavity is located almost diametrically opposite the ADPR binding cleft within which additional difference density consistent with adenine binding was observed on soaking crystals in ADPR, ADP and adenosine (Figure S3 and data not shown). The residues surrounding this pocket are highly conserved amongst mammalian PARGs, but lie in a region absent from both protozoal and bacterial PARG (Figure S1). We speculate that this pocket may correspond to a lower affinity secondary site for binding of adenine within an extended PAR chain. Given the significant separation of this additional site from the catalytic site, the design of targeted inhibitors via a linking strategy appears to have limited scope.

Consistent with the existence of additional ADPR binding pockets on the PARG surface, Shirato and co-workers [49] have shown that poly (etheno-ADPR) can inhibit PARG activity towards unmodified PAR without itself being hydrolysed. Etheno-derivatisation of the adenine moiety, however, appears incompatible with binding at both the ADPR sites identified in this work, due to the requirement for an intact H-bond donor at the 6-position of the adenine ring. Similarly, we were able to show binding of several AMP derivatives with substitutions at the 2- and 6- positions that would not be tolerated in either the primary or secondary ADPR pocket. Binding of these AMP derivatives to PARG was non-competitive with ADP-HPD as monitored by ligand-observed NMR (data not shown), suggesting the existence of additional binding pockets on the protein surface.

### Structure of hPARG26 in complex with ADP-HPD

ADP-HPD is a transition-state mimetic that shows competitive inhibition of PARG activity *in vitro* [38]. The HPD moiety of ADP-HPD mimics the proposed oxocarbenium ion intermediate of the glycohydrolysis reaction having a positive charge on the HPD nitrogen. Our structure shows ADP-HPD binds to the same site on PARG as ADPR, placing the HPD moiety in the catalytic site (Figure 4b), as also observed for rPARG [25] and TcPARG [23].

The ADPR and ADP-HPD complex structures superpose almost exactly (r.m.s. deviation 0.14 Å over all Cα; 0.47 Å over all atoms within the binding pocket (MOE, Chemical Computing Group)). ADPR and ADP-HPD engage in equivalent interactions, with the exception of loss of an H-bond between the 1″-OH and Glu756 as this OH is not present in ADP-HPD. ADP-HPD binds to hPARG with nanomolar affinity (Table 1), whereas ADPR binds with ∼1000-fold weaker affinity, such that we struggled to measure a reliable K_D_. SPR competition experiments, in which hPARG4-ADP-HPD complex was formed *in situ* on the sensor chip surface by flowing over a buffer containing a fixed, saturating concentration of ADP-HPD, then following this with increasing concentrations of ADPR, gave a K_D_ ∼40 µM (data not shown), which is consistent with the reported value for bPARG of 120 µM [38]. We speculate that the bound conformation of ADP-HPD is stabilised relative to ADPR by dipole-dipole interactions between the HPD NH_2_^+^ and a negatively charged α-phosphate oxygen and/or the oxygen linking the β-phosphate to ribose” (4.1 Å and 2.9 Å separation respectively, Figure 4b). In addition, the α-phosphate oxygen H-bonding network may be strengthened in the ADP-HPD complex by the charged interaction with the pyrrolidine NH_2_^+^. This oxygen accepts H-bonds from the backbone NH of Gln754 and a conserved water molecule, and is shielded from solvent by Val753. Binding of ADP-HPD is primarily enthalpy driven (Table 1). Consistent with this result, the majority of polar atoms are involved in H-bonds to either protein or water molecules (Figure 4b, 21 H-bonds reported by MOE (Chemical Computing Group)).

### Structure of hPARG26 in complex with OA-ADP-HPD

The 8-*n*-octylamino derivative of ADP-HPD (OA-ADP-HPD) has been reported as a cell permeable inhibitor of PARG [37]. In our hands, we were unable to show cell activity (data not shown). OA-ADP-HPD proved a more complete inhibitor of hPARG activity *in vitro* than ADP-HPD and was therefore used as a maximally-inhibited control in our High Throughput Screening (HTS) assay (Bennett et al, manuscript in preparation). In the ADPR and ADP-HPD complex structures, the 8-position of the adenine ring is occluded by the side-chain of Tyr795 (Figures 4b and 5a). Indeed, the equivalent residue in bPARG, Tyr796, can be photo-labelled with 8-azido-ADP-HPD [37]. In order to understand the unexpected inhibitory activity of OA-ADP-HPD, we determined the structure of OA-ADP-HPD in complex with hPARG26 at 2.14 Å. This shows that the ADP-HPD core of OA-ADP-HPD binds in an identical way to that observed for ADP-HPD itself. The 8-*n*-octylamino substituent is then accommodated by displacement of the Tyr795 side-chain to access solvent (Figures 4c and 5c). Consistent with the requirement for additional induced fit, and consequent energy penalty for binding, we measured a reduced K_D_ for OA-ADP-HPD binding to hPARG4 compared to ADP-HPD (800 nM vs. 132 nM, Table 1 and Figure 2d). Similarly, OA-ADP-HPD showed a decrease in potency in our *in vitro* homogeneous time-resolved fluorescence (HTRF) assay compared to ADP-HPD (16.3 µM vs. 3 µM, Table 1), in line with the reduced binding affinity.

### Implications for the PARG catalytic mechanism

Our high resolution structures provide support for the catalytic mechanism proposed by Slade and co-workers [23] for TcPARG, demonstrating conservation of key water molecules amongst the unliganded, ADPR-bound and ADP-HPD-bound states (Figures 4a and 4b). Binding of an ADPR moiety within a PAR chain positions the *O*-glycosidic link in hydrogen bond contact with Glu756 constraining the ribose” to the α-anomer (Figure 4a). Glu756 (equivalent to TcPARG Glu115 and rPARG Glu752) would then protonate the (n-1) PAR ribose' 2′-OH (linear chain) or possibly instead a ribose” 2″-OH (branch point) leaving group (Figure 6). The putative positively-charged oxocarbenium ion intermediate could be stabilised by negative charge on an oxygen of the α-phosphate which is held 4.1 Å from the HPD NH_2_^+^. As observed in TcPARG, a tightly-bound water molecule (Wat1, blue in Figure 6, Figure 4b inset) is positioned, by interactions with the di-phosphate moiety and the backbone NH of Glu755 and Asp737, for activation by Glu756 to attack the anomeric carbon of the oxocarbenium, leading to release of products with an inverting mechanism (shorter and possibly also unbranched PAR chains, and ADP-β-ribose”). Wat1 is present in the unliganded, ADPR-bound and ADP-HPD-bound hPARG26 structures. A second water (Wat2, red in Figure 6, Figure 4b inset) is present in the unliganded and ADP-HPD-bound structures above the plane of the HPD ring, in a position approximating that of the ADPR 1″-OH. Wat2 is also within H-bond distance of Glu756 (Figure 4b inset), and would allow for completion of the catalytic cycle via a retaining mechanism, generating ADP-α-ribose”. Finally, flexibility in the Tyr795 side-chain, as demonstrated by the OA-ADP-HPD complex structure, suggests a mechanism for product release.

### Rationale for ADP-HPD Structure-Activity Relationships

The SAR of ADP-HPD has been extensively studied and published (see references [6], [23], [25], [37], [50]. Together, the hPARG26-ADP-HPD and OA-ADP-HPD complex structures account for this SAR and the effects of site directed mutations on binding of ADP-HPD derivatives [6], [37], which is summarized in Table S1. By and large, these are as previously described [6], [37], [50], with additional, new observations concerning the impact of flexibility in the Tyr-795 side-chain.

Key H-bond interactions in the adenine and HPD pockets, combined with steric restrictions, constrain substitution at the 2- and 6-positions of the adenine moiety and C5 of the HPD moiety, and account for the loss of affinity previously reported for *des* 3- and 4-OH analogues of ADP-HPD [37]. Curiously, mutation of Glu728 to Asn in bPARG (equivalent to E727N in hPARG) was reported to have a limited impact on binding affinity for 8-AH-ADP-HPD as determined by SPR using immobilized 8-AH-ADP-HPD (K_D_ 8.2 nM vs. 3.2 nM for wild-type and mutant bPARG, respectively [6]), despite reducing catalytic activity to <20%. This suggests the shape of the adenine binding pocket is not significantly altered upon mutation of Glu727 to Asn. We speculate that changes in the H-bond network which links Glu727 with the adenine 6-NH_2_, Tyr792 and other residues in the ADPR binding site (e.g. Gln754) transmit to the catalytic centre to reduce activity. These changes may also impact on the flexibility of the “Tyr-clasp”, potentially reducing the penalty for displacement of Tyr795 and enhancing affinity for 8-AH-ADP-HPD.

Movement of Tyr795, as observed in the OA-ADP-HPD structure, creates additional space for substitution at the 8-position of the adenine ring, however, the channel remains somewhat constricted by the side-chain of Val753 (Figures 5a and 5c) thus accounting for the SAR at this position. Interestingly, mutation of Tyr796 to Ala in bPARG (equivalent to Tyr795Ala in hPARG) was observed to reduce affinity for 8-AH-ADP-HPD >20-fold [50]. Assuming a similar binding mode for 8-AH-ADP-HPD and OA-ADP-HPD, one might expect truncation of Tyr795 to Ala to facilitate binding by relieving the steric block imposed by Tyr795. The observed stacking interactions between the side-chain of Tyr795 and the 8-*n*-octylamino substituent of OA-ADP-HPD (Figures 4c and 5c) may account for the deleterious effect of the Tyr796Ala mutation and the lack of effect of the Tyr796Trp mutation [50].

In agreement with previous work [37], fragmentation of ADP-HPD resulted in a rapid drop off in potency. We measured an approximate K_D_ for AMP by SPR (0.38 mM, n = 1), using the same competition-based method as employed for ADPR. This showed a 10-fold drop-off in affinity upon removal of the β-phosphate-ribose moiety. Structures of hPARG26 in complex with adenine containing fragments suggested an intact diphosphate moiety was required to achieve complete closure of the phosphate binding loop and thus formation of the ribose”/HPD binding site (data not shown).

### Conclusion and Implications

We have used a battery of bioinformatic and experimental approaches to engineer a crystallisable fragment comprising the hPARG catalytic domain. Here, we report the first high resolution crystal structure of the hPARG catalytic domain in ligand-free form and in complex with ADPR and two PARG inhibitors, ADP-HPD and OA-ADP-HPD. Our structures confirm the conservation of the overall fold amongst mammalian PARG catalytic domains, as exemplified recently in the structures of rat and mouse PARG ([25] and **PDB ID: 4FC2**), whilst highlighting important similarities and differences between the bacterial and protozoal PARG structures [23], [24] and those of mammalian PARGs. Significantly, both the ribose' 2′-OH and ribose” 1″-OH are solvent exposed in the hPARG structure, which may allow binding of branched and linearly linked as well as terminal ADPR units within the PAR polymer, and thus provide an explanation for the endo- and exo-glycohydrolase activity previously reported for mammalian PARG. Additional differences between the bacterial, protozoal and human PARG structures, particularly around the adenine binding pocket, suggest that small molecule modulators of PARG activity will differ in their activity towards these forms of PARG. Our hPARG structures together rationalise the reported SAR for derivatives of the PARG inhibitor, ADP-HPD [37], [50].

A large body of data correlating site-specific mutations with PARG activity exists in the literature [5], [6], [23], [50]. Three aspects of the hPARG26 structure can account for the majority of these data (Figure 7 and Table S1). One subset of mutations lies within the catalytic site and negatively impacts catalytic activity and/or inhibitor binding (red and pink residues in the upper part of the lower panel in Figure 7). A second subset of mutations is located distal to the catalytic site and has no impact on activity (white in Figure 7). This includes the six surface entropy reduction mutations described herein. A third subset of mutations impacts on catalytic activity whilst being located distal to the catalytic site (red and pink residues in the lower part of the lower panel in Figure 7). Many of this third set would be predicted to disrupt the overall fold, hence accounting for their impact on PARG activity. Several mutations and deletions within the putative MTS (magenta ribbon in Figure 7) fall within this category. In particular, Leu471 and Leu474 pack on either side of Trp814 at the N-terminus of the “Tyr-clasp” [25] (cyan ribbon in Figure 7), holding this motif in place via formation of an H-bond between the intervening Pro472 and the Trp814 indole NH. As proposed by Kim and co-workers [25], the effects of these mutations on PARG activity most likely result from destabilization of the “Tyr clasp” motif. A fourth set of mutations have been reported to show enhanced catalytic activity (blue in Figure 7) and are more difficult to rationalize based on the structure of the isolated PARG catalytic domain. One can speculate that they might lead to relief from inhibition by the N-terminal regulatory domain or enhance PAR binding.

Our crystal system offers advantages over those of the closely related rat and mouse PARG, in that access to the active site is not restricted by crystal contacts and high resolution structures with inhibitors having a wide range of potencies can be readily obtained (data not shown). We have further demonstrated the utility of our hPARG catalytic fragment by developing SPR, ITC, NMR and high-throughput biochemical (HTRF) assays which we have used for the assessment of potential inhibitors identified in a high throughput screen of our corporate collection. We have exemplified three conformational states for the human PARG catalytic domain (unliganded, ADP-HPD-bound and OA-ADP-HPD bound) thus providing three high resolution models for use with computational methods of inhibitor design. Access to these protein structures enables structure-led design of, for example, fragment libraries (compounds of low molecular weight), which can feed into both virtual and *in vitro* screening efforts. Emerging data from such approaches can provide a more detailed mapping of the active site and, potentially, alternative binding sites such as that exemplified in Fig. S3. Ultimately, such knowledge will facilitate the design of bioisosteres of ADP-HPD and more drug-like inhibitors. Selective targeting of the unliganded state has been recently demonstrated by the structure of TtPARG in complex with the inhibitor, RBPI [24], and shows promise as a route to PARG inhibitors with more drug-like properties than ADP-HPD and its derivatives. Given the interest in PARG as a therapeutic target, we anticipate our findings will facilitate future studies and potentially development of small, cell-permeable inhibitors of PARG activity of benefit to cancer patients.

## Materials and Methods

### Reagents

Reagents were obtained from Sigma-Aldrich, unless otherwise specified. ADP-HPD was custom synthesized by Wuxi, Shanghai or purchased from Calbiochem. OA-ADP-HPD was synthesized according to the route described in Koh *et al*, 2003 [37].

### Protein analysis

Protein purity was estimated by SDS-PAGE on 5–12% BisTris-Tricine gels (Invitrogen) run in MES running buffer with SeeBluePlus2 molecular weight markers. Protein concentrations were estimated from OD_280_ measured using a NanoDrop, using extinction coefficients calculated with VectorNTi (Invitrogen). Intact protein molecular weights were determined by LC-ESI-MS. Chromatography was performed using an Agilent 1100 HPLC system fitted with a Phenomenex Gemini reversed-phase chromatography column (3 µm bead, C18, 110 Å pore, 2.0×30.0 mm). Pre-equilibrating the column in eluent A (97% water, 3% acetonitrile, 0.05% formic acid) prior to sample injection, elution was performed at 300 µL/min with a nonlinear gradient of 0–100% B (97% acetonitrile, 3% water, 0.05% formic acid) over 9.5 minutes. The column eluate was directed into the electrospray source of a Waters LCT (electrospray-time of flight) mass spectrometer and the data collected over the duration of chromatography (19 mins) between 100 and 2400 m/z. Mass spectrometric data were deconvoluted using the MaxEnt1 algorithm in the Waters MassLynx software. The system was externally calibrated using horse heart myoglobin as the standard.

### PARG Expression and Purification Screen

A total of 29 constructs were designed (see Figure 1), and the corresponding codon optimised genes were synthesised (GeneArt), cloned into pET28a to add an N-terminal TEV protease-cleavable 6His tag, and tested in a multiple-parallel fashion at small scale for soluble, purifiable expression in *E. coli*. Briefly, 3 mL Terrific Broth (TB) supplemented with kanamycin (50 µg/mL) and tetracycline (12.5 µg/mL) in 24-well deep-well blocks was inoculated in duplicate with overnight starter cultures and grown to an OD_600_ = 0.6–0.8 at 37°C prior to induction with 0.1 mM IPTG at 18°C. Growth was continued overnight and cultures were harvested by centrifugation. Lysis was effected by one cycle of freeze/thaw followed by agitation for 30 mins at room temperature in 0.5 mL binding buffer (40 mM HEPES pH 8.0, 0.3 M NaCl, 20 mM imidazole, 10% glycerol, 1 mM TCEP) supplemented with benzonase (Novagen, 5 U/mL), lysozyme (1 mg/mL) and protease inhibitors (Roche Complete™ EDTA-free protease inhibitor tablet), and a second cycle of freeze/thaw. Soluble His-tagged proteins were extracted from the clarified lysate by capture on Ni-IMAC 1000+ PhyTips™ using a Phynexus MEA personal purification system. Resin was pre-equilibrated and washed with binding buffer (2×1 mL), and bound proteins eluted with binding buffer supplemented with 0.5 M imidazole (0.255 mL). Yields of soluble protein were estimated from SDS-PAGE gels of the eluants. Expression of promising constructs was carried out at 7.2 L scale and these were purified for crystallisation screening (as described for individual constructs below).

### Limited Proteolysis

Limited proteolysis experiments were carried out with the aim of identifying stable fragments to guide the design of shorter constructs. Initially we used construct PARG2 (hPARG(406–976)) and tested three proteinases, Trypsin, Endoproteinase AspN and Endoproteinase GluC (all from Roche and made up at 0.5 mg/mL in either 1 mM HCl (Trypsin) or water), at four ratios of proteinase to PARG (1∶10, 1∶40, 1∶100, 1∶400), incubating at room temperature and sampling at four time points (15 minutes and 1, 3 and 16 hours). Promising conditions were confirmed using constructs PARG3 (hPARG(432–976)) and hPARG4 (hPARG(448–976)). Endoproteinase AspN and Endoproteinase GluC had little effect, whilst Trypsin showed reproducible cleavage of all three constructs tested, yielding four stable proteolytic fragments. We then used the following conditions to generate material for N-terminal sequencing and Mass Spectrometric analysis: hPARG4 (stock at 0.5 mg/mL in SEC buffer (50 mM HEPES, pH 7.0, 150 mM NaCl, 2 mM DTT)) was incubated with Trypsin (stock at 0.5 mg/mL in 1 mM HCl) at a ratio of 1∶10 Trypsin: hPARG4 for 1 hour at room temperature. Samples were split and half was subjected to separation on SDS-PAGE, blotted onto PVDF membrane and submitted for N-terminal sequencing. The remainder was submitted for ESI-LC-MS (see Protein Analysis). The most stable fragment had an intact mass of 37 264 Da, and an N-terminal sequence of SEYSSY, corresponding to residues 651 to 973. The remaining fragments corresponded to residues 448 to 650, 448 to 527 and 527 to 973.

### Purified human full-length PARG (hPARG) for SPR immobilisation and biochemical assay

Obtained as follows: a codon-optimized gene encoding human PARG(1–976) was synthesized by GeneArt and subcloned into pET29b(+) in order to direct expression of hPARG(1–976) with a C-terminal hexahistidine tag. Protein expression in *E. coli* BL21 (DE3) GOLD was induced by addition of 0.1 mM IPTG to a shake flask culture grown to OD_600_ = 0.3 at 37°C, and then grown to OD_600_ = 0.6 at 18°C in TB supplemented with kanamycin (50 µg/mL) and tetracycline (12.5 µg/mL). Growth was allowed to continue at 18°C for a further 22 hours before harvesting by centrifugation (12 000 g), and storage of the cell pellets at −80°C. Protein was purified by immobilised metal affinity chromatography (IMAC) and size exclusion chromatography (SEC): frozen cell pellets (typically 40 g wet weight) were resuspended by homogenization in 10 volumes buffer A (50 mM KH_2_PO_4_, pH 8.0, 400 mM KCl, 5 mM β-mercaptoethanol, 10% glycerol, 100 µM PMSF, 1 µg/mL pepstatin, 1 µg/mL leupeptin, 1 µg/mL aprotinin), supplemented with lysozyme (1.0 mg/mL) and benzonase (Novagen, 5 U/mL), and lysed by sonication. The lysate was clarified by centrifugation for 45 minutes at 25 000 g, 4°C. The lysate supernatant was then passed over an 8 mL HiTrap NiNTA column (QIAGEN) equilibrated with buffer A. The column was washed with buffer A, then buffer A supplemented with 25 mM imidazole (∼10 column volumes (CV)). Bound proteins were eluted with buffer A supplemented with 250 mM imidazole. Pooled fractions containing PARG(1–976)-6His were subjected to SEC on a 320 mL Superdex200 column (GE Healthcare), pre-equilibrated and run in buffer B (50 mM KH_2_PO_4_, pH 7.8, 400 mM KCl, 5 mM β-mercaptoethanol, 10% glycerol). IMAC and SEC were automated and injection, wash and elution steps performed on an ÄKTA™ purifier FPLC system (GE Healthcare). Pooled fractions containing PARG(1–976)-6His were snap frozen in liquid nitrogen and stored at −80°C. Typically, 1.2 mg purified hPARG was obtained per gramme cell paste.

### Purified human PARG catalytic domain (hPARG4) for biochemical assay, SPR immobilisation and ITC

Obtained as follows: a codon optimized gene encoding human PARG(448–976) was synthesized by GeneArt, and subcloned into pET28b (BamHI/XhoI) so as to direct expression of PARG(448–976) with an N-terminal, TEV protease-cleavable 6His tag. Protein expression in *E. coli* BL21 (DE3) GOLD was induced by addition of 0.1 mM IPTG to a shake flask culture grown to OD_600_ = 0.3 at 37°C, and then grown to OD_600_ = 0.6 at 18°C in Terrific Broth (TB) supplemented with kanamycin (50 µg/mL) and tetracycline (12.5 µg/mL). Growth was allowed to continue at 18°C for a further 22 hours before harvesting by centrifugation (10 500 g, 4°C), and storage of the cell pellets at −80°C.

Protein expression was also achieved at 20 L scale by fermentation, according to the following protocol. A 10 mL starter grow was prepared in Luria Bertani (LB) broth by shaking for 7 hours at 37°C. 1 mL of starter grow was then inoculated into 600 mL LB and growth continued overnight. The overnight growth was then used to inoculate 20 L HYE20 broth supplemented with kanamycin (50 µg/mL) and tetracycline (12.5 µg/mL) at 10°C in a Braun Biostat-C 30 L bioreactor. The reactor was warmed to 37°C, fed with yeast extract (Difco Beta Lab) at a rate of 250 g/hour up to a total of 225 g/L culture, and growth continued until an OD_550_ = 15–20 was reached (typically 5–6 hours). Protein expression was then induced by the addition of 0.1 mM IPTG. Growth was continued for 22 hours at 18°C, feeding with yeast extract at a rate of 125 g/hour and, from 9½ hours post-inoculation, Carbon/Nitrogen feed (55% w/w glycerol, 11% w/w ammonium sulphate) at a rate of 150 g/hour.

Protein was purified by IMAC: frozen cell pellets (typically 200 g wet weight) were resuspended by homogenization in 10 volumes phosphate buffered saline (PBS, pH 7.4), supplemented with 5 mM β-mercaptoethanol and protease inhibitors (Roche Complete™ EDTA-free protease inhibitor tablet), and incubated for 15 minutes on ice before completion of lysis either by passage through a Constant Systems BasicZ homogenizer (larger volumes) or by sonication (smaller volumes). When sonication was used for cell lysis, the lysis buffer was supplemented with 0.2 mg/mL lysozyme. The lysate was clarified by centrifugation for 45 minutes at 25 000 g, 4°C, and the lysate supernatant incubated at 4°C for 30 minutes with NiNTA beads (QIAGEN) (typically 5–7.5 mL beads per 1 L cleared lysate) equilibrated with buffer C (20 mM Tris/HCl, pH 8.0, 400 mM NaCl, 5 mM β-mercaptoethanol, 5 mM imidazole). The beads were loaded into a gravity flow column (BioRAD Econocolumn), washed with Buffer C, then Buffer C containing 20 mM imidazole, and bound proteins eluted with Buffer C containing 200 mM imidazole. Pooled fractions containing 6His-TEV-PARG(448–976) were incubated with 6His-tagged TEV protease whilst being dialysed against Buffer C overnight at 4°C. GS-PARG(448–976) was separated from uncleaved material, 6His tag and TEV protease by subtractive NiNTA chromatography. The unbound material was concentrated to ∼2–5 mg/mL in a stirred cell using a YM10 membrane (Amicon), and then dialysed against two changes of SEC buffer. Typically, 0.2 mg purified hPARG4 was obtained per gramme cell paste.

### Purified human PARG catalytic domain surface entropy mutant (hPARG26) for crystallisation

Obtained as follows: a codon optimized gene encoding human PARG(448–976 [K616A, Q617A, K618A, E688A, K689A, K690A]) was synthesized by GeneArt, and subcloned into pET28b (BamHI/XhoI) so as to direct expression of PARG(448–976 [K616A, Q617A, K618A, E688A, K689A, K690A]) with an N-terminal, TEV protease-cleavable 6His tag. Unlabelled protein expression was carried out in shake flasks as described for hPARG4. Expression of SeMet labeled protein in the Met auxotroph *E. coli* B834 (DE3) was induced by addition of 1 mM IPTG and 0.2% w/v glucose to a shake flask culture grown to OD_600_ = 0.3 at 37°C, and then grown at 18°C until reaching an OD_600_∼0.6 in M9 minimal medium supplemented with selenomethionine (50 mg/L) and kanamycin (50 µg/mL). Growth was allowed to continue at 18°C for a further 20 hours before harvesting by centrifugation (10 500 g, 4°C), and storage of the cell pellet at −80°C.

Unlabelled protein was purified by IMAC and SEC: frozen cell pellets (typically 60–90 g wet weight, ∼3 L culture) were resuspended by homogenization in 10 volumes buffer D (50 mM Tris/HCl pH 8.0, 400 mM NaCl, 5 mM β-mercaptoethanol, 5 mM imidazole), supplemented with 0.3 mg/mL Lysozyme, 2.5 U/mL Benzonase (Novagen) and protease inhibitors (Roche Complete™ EDTA-free protease inhibitor tablet), and lysed by passage through a Constant Systems BasicZ homogenizer. The lysate was clarified by centrifugation for 45–60 minutes at 25 000 g, 4°C, and the lysate supernatant incubated at 4°C for 30 minutes with NiNTA beads (QIAGEN) equilibrated with buffer D (typically 5 mL beads per 0.8–1 L cleared lysate). The beads were loaded into a gravity flow column, washed with lysis buffer (∼10 CV), then buffer D containing 20 mM imidazole (∼10 CV), and bound proteins eluted with lysis buffer containing 200 mM imidazole. The unbound fraction was then incubated with a fresh batch of pre-equilibrated NiNTA beads, washed and bound protein eluted as before. Pooled fractions containing 6His-TEV-hPARG26 were incubated with 6His-tagged TEV protease whilst being dialysed against buffer D overnight at 4°C. GS-hPARG26 was separated from uncleaved material, 6His tag and TEV protease by subtractive NiNTA chromatography. The unbound material was concentrated in a stirred cell using a 10 k MWCO YM10 membrane (Amicon) and 2 mL concentrated sample was filtered (0.22 µm or 0.45 µm) before loading on a 125 mL Superdex75 sizing column (GE Healthcare) pre-equilibrated with SEC buffer. Pooled fractions containing GS-hPARG26 were concentrated using a 10 k MWCO spin concentrator (VivaSpin) to 10 mg/mL, and then either used immediately for crystallisation or snap-frozen in liquid nitrogen for storage at −80°C. Typically, <0.05 mg purified hPARG26 was obtained per gramme cell paste.

SeMet labeled protein was purified by IMAC and SEC essentially as described for unlabelled protein, with the following minor alterations: 17.5 g cell paste (∼7.2 L culture) were lysed, and a single capture step, using 3 mL NiNTA beads per 200 mL cleared lysate was employed. <0.05 mg purified SeMet labelled hPARG26 was obtained per gramme cell paste.

### Immobilisation of recombinant hPARG, hPARG4 and hPARG26 for SPR studies

Biosensor (SPR) analyses were conducted using a BIAcore 3000 or BIAcore S51 instrument. Research grade CM5 chips and coupling reagents (N-ethyl-*N′*-dimethylaminopropylcarbodiimide, EDC; *N*-hydroxysuccinimide, NHS; and 1 M ethanolamine HCl, pH 8.5) were purchased from BIAcore (GE Healthcare, Northampton, MA).

A CM5 chip was docked into the instrument, primed 5 times with filtered and degassed running buffer containing 50 mM HEPES pH 7, followed by preconditioning at 100 µL/min using two consecutive aliquots of 50 µL each of 10 mM HCl, 50 mM NaOH, 0.1% (w/v) SDS, 0.085% (v/v) H_3_PO_4_. PARG surfaces were prepared by standard amine coupling via exposed amines on PARG. Immobilisations were conducted at 25°C in 50 mM HEPES at a flow rate of 5 µL/min. Flow cells were activated for 7 min by injecting a 35 µL mixture of 50 mM NHS: 200 mM EDC. Subsequently, 35 µL of 100 µg/mL PARG was injected for 7 min, followed by a 35 µL injection of ethanolamine. Typical immobilisation levels ranged from 3000 to 5000 resonance units (RU). Nonderivatised flow cells served as reference surfaces.

### Kinetic interaction studies

Studies of PARG inhibitor binding were conducted at 25°C. Samples were prepared as 5 fold dilutions in the experimental running buffer (20 mM HEPES pH 7, 150 mM NaCl, 2 mM DTT, 0.005% P20, 0.02% NaN_3_ with 5% DMSO). Surface regeneration was achieved using dissociation for a time period allowing the response to return to baseline. Control injections of a fixed, saturating ADP-HPD concentration of 20 µM were interspersed with injections of compound to allow monitoring of the functionality of the protein surface. To calculate affinities, SPR equilibrium binding data, consisting of Req values from 8–10 point concentration series, were analyzed by fitting a simple 1∶1 binding model to yield Rmax and Kd values using non-linear regression analysis in Grafit 6 (Erithacus Software).

### Isothermal titration calorimetry

This was carried out using an iTC200 instrument (Microcal, GE Healthcare, Northampton, MA) at 25°C in the following buffer: 20 mM HEPES pH 7, 150 mM NaCl, 2 mM DTT, 0.02% NaN_3_. Protein concentrations were typically 30 µM in the cell with ligand concentrations of at least 10 fold this concentration in the syringe. Typical injection protocols of 19×2 µL injections, spaced at 2 or 3 minute intervals were used. Curves were fitted by non-linear regression analysis using a one-site binding model provided by MicroCal Origin software (version 7).

### PARG activity assay

PARG activity was assessed using an HTRF assay which will be described in detail elsewhere (Bennett et al, manuscript in preparation). Briefly, biotin-labelled PARylated PARP1 substrate was prepared as follows: PARP1 was incubated in the presence of double stranded DNA prepared by annealing complementary oligonucleotides (as a surrogate for DNA damage and hence activation of PARP1) and nicotinamide adenine dinucleotide (NAD^+^) at a 32∶1 molar ratio of NAD^+^:PARP1 for 2 hours. Following this incubation, biotinylated NAD^+^ at a molar ratio to PARylated PARP1 of 0.7∶1 was added and incubation continued for 5 minutes. The reaction was terminated by addition of a PARP1-specific inhibitor to a final concentration of 4.7 µM. The solution was dialysed overnight to remove excess biotinylated NAD^+^ and compound, and stored at −80°C. PARG activity was measured in a 384-well format HTRF assay using the biotin-labelled PARylated PARP1 as a substrate. Residual biotinylated PARylated PARP1 was detected by addition of a detection mix containing anti-6His–XL antibody (Cisbio) and streptavidin europium cryptate (Cisbio), and then monitoring the ratio of emission at 665 nm to 612 nm upon excitation at 340 nm.

Inhibition by ADP-HPD was measured using serial dilutions over the range 100 µM to 100 pM compound from a 1 mM stock in water. Assays contained 30 pM purified recombinant PARG and 9.67 nM PAR-PARP1 in a final volume of 9 µL assay buffer (50 mM MOPS pH 7.4, 0.1 mg/mL BSA, 3 mM EDTA, 0.4 mM EGTA, 1 mM DTT, 0.01% v/v Tween20, 50 mM KCl). Reactions were allowed to proceed for 8 minutes prior to the addition of 3 µL detection reagents (50 mM MOPS pH 7.4, 0.1 mg/mL BSA, 0.1 M Potassium Fluoride, 14 nM anti-6HIS-XL antibody, 0.75 nM Streptavidin Europium Cryptate). IC_50_s were calculated using a time-point of 1.5 hours from the addition of detection reagents. Under these conditions we measured a higher IC_50_ for ADP-HPD towards hPARG than under the conditions used in our High Throughput Screening (HTS) Campaign, where we routinely obtained an IC_50_ in the region of 600 nM.

IC_50_ determination for OA-ADP-HPD was carried out as part of an HTS Campaign where the HTRF assay was scaled down to a 3 µL reaction to which 1.5 µL detection reagents were added after the reaction had been allowed to proceed for 10 minutes. Here, 17.5 nL of ADP-HPD (60 µM in DMSO) or OA-ADP-HPD (21.43 mM in DMSO) were dispensed by an ECHO555 (Labcyte Inc.) into Greiner white 1536-well plates over the specified range: ADP-HPD (100 µM to 100 pM) and OA-ADP-HPD (500 µM to 30 nM).

ADP-HPD and OA-ADP-HPD inhibition data were visualised in Prism (GraphPad), and IC_50_s derived from a non-linear regression analysis of the values from three separate experiments (run in triplicate) fit to a sigmoidal dose-response curve.

### Protein Crystallisation

Crystals of SeMet labelled GS-hPARG26 were grown at 293 K by sitting-drop vapour diffusion by mixing purified protein in SEC buffer at 7.5 mg/mL with a precipitant consisting of 28% PEG-3350, 0.2 M magnesium chloride, 0.1 M PCTP (0.04 M sodium propionate, 0.02 M sodium cacodylate, 0.04 M Bis-Tris propane) pH 7.5 in a 1∶1 ratio to give a 4 µL drop. Crystals appeared within 4 days and continued to grow for a further two weeks. Crystals were passed quickly through a cryoprotectant buffer (25% PEG-3350, 0.2 M ammonium sulphate, 0.1 M PCTP pH 7.5, 10% glycerol) then flash cooled in a gaseous nitrogen stream at 100 K prior to data collection. SeMet labelled GS-hPARG26 crystals belonged to the monoclinic space group, P2_1_, with unit cell dimensions 44.7×66.4×89.3 Å, β = 95.2°. The asymmetric unit comprises a monomer.

Crystals of unlabelled human GS-hPARG26 were grown at 293 K by sitting-drop vapour diffusion by mixing purified protein at 7.5 mg/mL in SEC buffer with a precipitant consisting of 18–23% PEG-3350, 0.2 M ammonium sulphate, 0.1 M PCTP pH 7.5 in a 1∶1 ratio to give a 4 µL drop. Crystals appeared overnight and continued to grow for a further week. Crystals were passed quickly through a cryoprotectant buffer (25% PEG-3350, 0.2 M ammonium sulphate, 0.1 M PCTP pH 7.5, 10% glycerol) then flash cooled in a gaseous nitrogen stream at 100 K prior to data collection.

Complex structures were obtained by incubating crystals of unlabelled GS-hPARG26 for periods of 16 hours to 7 days in a soak buffer (25% PEG-3350, 0.2 M ammonium sulphate, 0.1 M PCTP pH 7.5–8.5, 10% glycerol) containing the compound of interest (5–10 mM) and ≤20% DMSO. Crystals were then flash cooled in a gaseous nitrogen stream at 100 K prior to data collection. Unlabelled GS-hPARG26 crystals belonged to the orthorhombic space group, P2_1_2_1_2_1_, with unit cell dimensions 66.9±0.1×90.6±0.2×94.8±0.4 Å. The asymmetric unit comprises a monomer.

### X-ray Diffraction Data Collection, Structure Solution and Refinement

Attempts to soak a variety of heavy atoms into the hPARG26 crystals were unsuccessful, resulting in severely degraded diffraction quality. Soaks with high concentrations of NaBr or MagicTriangle [51] did not yield useful phase information. We were unable to use the intrinsic sulphur anomalous signal to derive phasing information, possibly as a result of the absence of disulphide bridges, despite the presence of 14 cysteines within the sequence. We therefore expressed, purified and crystallised SeMet labelled protein to enable structure solution by the MAD method. X-ray diffraction data were collected at 3 and 4 wavelengths around the Se edge on beam line ID23-EH1 at the ESRF from two crystals of SeMet labelled hPARG26. The better anomalous signal was obtained from the crystal in which the SeMets were partially oxidised, as evidenced by the X-ray fluorescence spectrum, and these data were used for structure solution, however, this dataset was somewhat compromised by ice rings yielding an effective resolution of 2.4 Å. Data integration, space group determination, scaling and data reduction were carried out using XDS [52], pointless, scala and truncate [53], [54] as implemented within autoPROC [55]. Data collection statistics are given in Table 2. 10 of 11 SeMet positions were located and refined, and initial phases calculated using the ShelX suite [56] as implemented within CCP4i [57]. An initial model was auto-built into the ShelxE maps using ARP/wARP [58]. Additional phase refinement, density modification and automated model building were carried out using Buccaneer [59] and Parrot [60], and subsequently also SHARP [61], SOLOMON [62] and ARP/wARP [58] as implemented within autoSHARP [63]. All three partial models were combined and model completion was carried out manually in Coot [64] using maps from all three phasing routes. Cross-correlation of the maps and models was particularly helpful in building more flexible regions of the structure. Refinement was completed against the 1.83 Å resolution inflection point dataset collected from the second SeMet hPARG26 crystal. The final model, comprising residues 450 to 523 and 530 to 963, was refined using Refmac [65], [66]. Quality checks were carried out using the validation tools within Coot [64] and MolProbity [67]. Crystallographic statistics indicating data and model stereochemical quality are given in Table 2.

Comparison of the hPARG26 structure with other structures in the PDB at the time this structure was solved highlighted structural similarity between a highly conserved stretch of ∼200 amino acids in the C-terminal portion of the PARG catalytic domain and an ADP-ribose binding macro domain from *Archaeoglobus fulgidus*, Af1521 [39]; **PDB ID: 2BFQ**, DALI Z score = 11.0, r.m.s. deviation = 2.7 Å for 165 Cα atoms, corresponding to residues 716 to 922 of hPARG26. Subsequent depositions reveal significant structural similarity with *Thermonospora curvata* PARG (**PDB ID: 2SIJ**, DALI Z score = 14, r.m.s. deviation = 3.3 Å for 265 Cα atoms), *Tetrahymena thermophila* PARG [24] (**PDB ID: 4EPQ**, DALI Z score = 37, r.m.s. deviation = 2.6 Å for 439 Cα atoms) and the catalytic domains of rat [25] and murine PARG (**PDB ID: 3UEK**, DALI Z score = 60.5, r.m.s. deviation = 0.6 Å for 521 Cα atoms, **PDB ID: 4FC2**, DALI Z score = 62.1, r.m.s. deviation = 0.6 Å for 505 Cα atoms).

Diffraction data for crystals of unlabelled hPARG26 were collected at 100 K using either a Rigaku FRE rotating anode X-ray generator equipped with VariMaxHF optics and a Saturn944 CCD detector or on beam line ID29 at the ESRF (see Table 3). Data processing was carried out using MOSFLM [68], d*TREK [69] or XDS [52] as implemented within autoPROC [55]. Data reduction and structure solution by molecular replacement (initially using the monoclinic SeMet-hPARG26 structure as a starting model, and later using the higher resolution ligand-free orthorhombic hPARG26 structure) were carried out using programs from the CCP4 suite [70]. Compounds were modeled into the electron density using Flynn as implemented within AFITT [71] (version 2.0.1, OpenEye Scientific Software, Inc., Santa Fe, NM, USA. OpenEye Scientific Software website. Available: www.eyesopen.com. Accessed 13^th^ November 2012). The protein-compound complex model was refined using Refmac [65], [66], [72] and/or Buster [73] (Global Phasing Ltd., Cambridge, UK) with intermediate rounds of model building in Coot [64]. The final structures have been deposited in the Protein Data Bank together with structure factors and detailed experimental conditions (see Table 3 for crystallographic statistics and PDB accession codes).

### Molecular Modelling

In order to assess the effects of site-directed mutations on the hPARG26-ADP-HPD structure, residues were first individually mutated in Coot [64], selecting a side-chain rotamer that minimized steric clashes. Mutated coordinates for the hPARG26-ADP-HPD complex, including crystallographic water molecules were subjected to the Structure Preparation, Protonate3D and Energy Minimisation routines in MOE (Chemical Computing Group), using default parameters. The output was visually compared to the wild-type structure.

## Supporting Information

Figure S1 **Structure based alignment of PARG sequences from mammals, plants, protozoa and bacteria against the Af1521 macrodomain sequence highlights areas of conservation around the ADPR binding site.** (Hs = *Homo sapiens*, Bt = *Bos taurus*, Mm = *Mus musculus*, Rn = *Rattus norvegicus*, At = *Arabidopsis thaliana*, Tc = *Thermonospora curvata*, Tt = *Tetrahymena thermophila*) Sequences were extracted from the PDB, or from UniProt where a structure was not available (Bt & At), and aligned on the basis of structure and sequence using the Superpose Ligands and SSM Superpose features in Coot and the Align and Superpose features in MOE (Chemical Computing Group). The alignment was visualised, edited and coloured according to sequence similarity using VectorNTI (Invitrogen). Key: Mutations described in Table S1 are indicated above the sequence with a circle coloured according to their effect on PARG activity from red (activity abolished) through grey (no effect) to blue (activity enhanced). Residues within 3.5 Å of bound ADP-HPD are indicated above the sequence with a filled triangle. Residues within 3.5 Å of the secondary adenine binding pocket are indicated above the sequence with an open triangle. Conserved motifs and the N-and C-terminal extents of the macro-domain core are labelled above the sequence and indicated with a coloured line. Secondary structural elements in hPARG26 are shown in schematic form above the sequence as follows; cylinder = α-helix, arrow = β-sheet, dotted line = disordered region. Residues missing from the co-ordinates used to generate the alignment are indicated in italics.
(PDF)

Figure S2 **Representative binding isotherms for binding of ADP-HPD to hPARG as measured by ITC.** The thermodynamic values extracted from these data were heavily influenced by noise in the base-line. The curve shown in the figure is fit to values derived from the mean of two methods of base-line calculation.
(TIF)

Figure S3 **A second adenine binding pocket lies on the opposite face to the ADPR binding cleft.** Bound ADPR and Adenine fragment are shown in spheres with carbon atoms in grey. Inset shows details of the secondary adenine binding site with adenine and selected PARG residues in stick representation (carbons in grey). Final 2Fo-Fc electron density for bound adenine is shown contoured at 1σ.
(TIF)

Table S1 **Mapping PARG mutational data onto the hPARG26 structure.**
(DOCX)
