# Supplementary material for: Structures of the Human Poly (ADP-Ribose) Glycohydrolase Catalytic Domain Confirm Catalytic Mechanism and Explain Inhibition by ADP-HPD Derivatives
Source: PLoS One. 2012 Dec 10;7(12):e50889. doi: 10.1371/journal.pone.0050889 (PMC3519477; doi:10.1371/journal.pone.0050889)
Supplement: Figure S1 — Structure based alignment of PARG sequences from mammals, plants, protozoa and bacteria against the Af1521 macrodomain sequence highlights areas of conservation around the ADPR binding site. (Hs = Homo sapiens, Bt = Bos taurus, Mm = Mus musculus, Rn = Rattus norvegicus, At = Arabidopsis thaliana, Tc = Thermonospora curvata, Tt = Tetrahymena thermophila) Sequences were extracted from the PDB, or from UniProt where a structure was not available (Bt & At), and aligned on the basis of structure and sequence using the Superpose Ligands and SSM Superpose features in Coot and the Align and Superpose features in MOE (Chemical Computing Group). The alignment was visualised, edited and coloured according to sequence similarity using VectorNTI (Invitrogen). Key: Mutations described in Table S1 are indicated above the sequence with a circle coloured according to their effect on PARG activity from red (activity abolished) through grey (no effect) to blue (activity enhanced). Residues within 3.5 Å of bound ADP-HPD are indicated above the sequence with a filled triangle. Residues within 3.5 Å of the secondary adenine binding pocket are indicated above the sequence with an open triangle. Conserved motifs and the N-and C-terminal extents of the macro-domain core are labelled above the sequence and indicated with a coloured line. Secondary structural elements in hPARG26 are shown in schematic form above the sequence as follows; cylinder = α-helix, arrow = β-sheet, dotted line = disordered region. Residues missing from the co-ordinates used to generate the alignment are indicated in italics. (PDF) [file pone.0050889.s001.pdf]

```
                    MTS
               448                                                                       520
hPARG26   (448) --SPDKKWLGTPIEEMRRMPRCGIRLPLLRPSANHTVTIRVDLLRAGEVPKPFPTHYKDLWDNKHVKMPCSEQNL
 BtPARG   (447) HLSPDKKWLGTPIEEMRRMPRCGIRLPPLRPSANHTVTIRVDLLRIGEVPKPFPTHFKDLWDNKHVKMPCSEQNL
 RnPARG   (442) NLPPDKKWLGTPIEEMRKMPRCGVRLPLLRPSASHTVTVRVDLLRAGEVPKPFPTHYKDLWDNKHVKMPCSEQNL
 MmPARG   (439) NLPPEKKWLGTPIEEMRKMPRCGIHLPSLRPSASHTVTVRVDLLRAGEVPKPFPTHYKDLWDNKHVKMPCSEQNL
 AtPARG     (1) -------------------------------------MENREDLNSILPYLPLVIRSSSLYWPPRVVEALKAMSE
 TtPARG     (1) -------------------------------------------------MIEAEKQENKKIQDYQFPLPQ-----
 TcPARG     (3) ---------------------------------------------------------------------------
 Af1521     (1) ---------------------------------------------------------------------------

               521                                                                       595
hPARG26   (521) YPVEDENGERTAGSRWELIQTALLNKFTRPQNLKDAILKYNVAYSKKWDFTALIDFWDKVLEEAEAQHLYQSILP
 BtPARG   (522) YPVEDENGERAAGSRWELIQTALLNRLTRPQNLKDAILKYNVAYSKKWDFTALIDFWDKVLEEAEAQHLYQSILP
 RnPARG   (517) YPVEDENGERTAGSRWELIQTALLNKFTRPQNLKDAILKYNVAYSKKWDFTALVDFWDKVLEEAEAQHLYQSILP
 MmPARG   (514) YPVEDENGERTAGSRWELIQTALLNKFTRPQNLKDAILKYNVAYSKKWDFTALVDFWDKVLEEAEAQHLYQSILP
 AtPARG    (39) GPSHS-QVDSGEVLRQAIFDMRRSLSFSTLE--PSASNGYAFLFDELIDEKESKRWFDEIIPALASLLLQFPSLL
 TtPARG    (21) -KNSELWIIQKKTLQDLSSGKQKLDSFQSLESILEILRDSK---NQNDEKYFNLKAVFEQLDKEEQTYFLEQFIP
 TcPARG     (3) ---------------------------------------------------------------------------
 Af1521     (1) ---------------------------------------------------------------------------

               596                                                                       669
hPARG26   (596) DMVKIALCLPNICTQP-IPLLAAAMNHSITMSQEQIASLLANAFFCTFPRRNAKMKSEYSSYPDINFNRLFEGRS
 BtPARG   (597) DMVKIALCLPNICTQP-IPLLKQKMNHSITMSQEQIASLLANAFFCTFPRRNAKMKSEYSSYPDINFNRLFEGRS
 RnPARG   (592) DMVKIALCLPNICTQP-IPLLKQKMNHSVTMSQEQIASLLANAFFCTFPRRNAKMKSEYSSYPDINFNRLFEGRS
 MmPARG   (589) DMVKIALCLPNICTQP-IPLLKQKMNHSVTMSQEQIASLLANAFFCTFPRRNAKMKSEYSSYPDINFNRLFEGRS
 AtPARG   (111) EVHFQNADNIVSGIKTGLRLLNSQQAGIVFLSQELIGALLACSFFCLFPDDNRGAKHLPVINFDHLFASLYISYS
 TtPARG    (93) KICQLVLKIK--KKQLKNQIPKESKIYEAAFSREEISYYVSCMFLCILKDQDRKIYKDFRLIYLKDLVQQINIRR
 TcPARG     (3) -------------------------------------------------------------------RHSRRAIAAE
 Af1521     (1) ---------------------------------------------------------------------------

                                                                              Macrodomain
               670                                                                       730
hPARG26   (670) SRKPEKLKTLFCYFRRVTAAAPTGLVTF--TRQSLEDFP----EWERCEKPL--------TRLHVTYEGTIEENG
 BtPARG   (671) SRKPEKLKTLFCYFRRVTEKKPTGLVTF--TRQSLEDFP----EWERCEKLL--------TRLHVTYEGTIEGNG
 RnPARG   (666) SRKPEKLKTLFCYFRRVTEKKPTGLVTF--TRQSLEDFP----EWERCDKPL--------TRLHVTYEGTIEGNG
 MmPARG   (663) SRKPEKLKTLFCYFRRVTEKKPTGLVTF--TRQSLEDFP----EWERCEKPL--------TRLHVTYEGTIEGNG
 AtPARG   (186) QSQESKIRCIMHYFERFCSCVPIGIVSFERKITAAPDAD----FWSKSDVSL--------CAFKVHSFGLIEDQP
 TtPARG   (166) QEKIKCFYEYLKQALDFSEKESKEVVIF-QRINCGQLEDYENWVDKLKAIKL--------KNVQLTDDKLIEDFP
 TcPARG    (13) TVEILERGRYTAPSGRVVPIAHDVAQAVRGTRLYR---P---EKLAVLLEGLGAASDGAPTRIEVTE-ETTLAAA
 Af1521     (1) -------------------------------------------MEVLFEAKVGD---------ITLKLAQ-GDITQYP

                                  Catalytic loop
               731                                                                       785
hPARG26   (731) Q--------GMLQVDFANRFV-GGGVTSAGLVQEEIRFLINPELIISRLFTEV-----------LDHNECLIITG
 BtPARG   (732) Q--------GMLQVDFANRFV-GGGVTSAGLVQEEIRFLINPELIVSRLFTEV-----------LDHNECLIITG
 RnPARG   (727) R--------GMLQVDFANRFV-GGGVTGAGLVQEEIRFLINPELIVSRLFTEV-----------LDHNECLIITG
 MmPARG   (724) R--------GMLQVDFANRFV-GGGVTGAGLVQEEIRFLINPELIVSRLFTEV-----------LDHNECLIITG
 AtPARG   (249) D--------NALEVDFANKYL-GGGSLSRGCVQEEIRFMINPELIAGMLFLPR-----------MDDNEAIEIVG
 TtPARG   (232) ---------GTLQVDFANCDI-GGGILGNGLVQEEIRFCVCPEMLVSLLVFEQS----------MEANEVIIMKG
 TcPARG    (81) RRLTGAAGDQVACLNFASAEHPGGGFLSGAHAQEEGLARSSGLYASLRAVPQFYAFHHRQRDPLYSDHLIYSPGV
 Af1521    (26) A--------KAIVNAANKRLEHGGG-------VAYAIAKACAGDAGLYTEISKKAMREQFGRDYIDHGEVVVTPA

                                                 "Tyr-clasp"
               786                                                                       851
hPARG26   (786) TEQYSEYTGYAETYRWSRSH-------EDGSERDDWQRRCTEIVAIDALHFR--RYLDQFVPEKMRRELNKAYCG
 BtPARG   (787) TEQYSEYTGYAETYRWARSH-------EDRSERDDWQRRTTEIVAIDALHFR--RYLDQFVPEKIRRELNKAYCG
 RnPARG   (782) TEQYSEYTGYAETYRWARSH-------EDGSEKDDWQRCCTEIVAIDALHFR--RYLDQFVPEKVRRELNKAYCG
 MmPARG   (779) TEQYSEYTGYAETYRWARSH-------EDGSEKDDWQRRCTEIVAIDALHFR--RYLDQFVPEKVRRELNKAYCG
 AtPARG   (304) AERFSCYTGYASSFRFAGEY-------IDKKAMDPFKRRRTRIVAIDALCTP---KMRHFKDICLLREINKALCG
 TtPARG   (287) IKQYSDYQGYSNSFRFVKMGNSKIQKQKRNNP--------QTILAIDALCFN---SSDNQFSEVNVSRELNKSYM
 TcPARG   (156) PVFRDDA-----------GR-------LLEEP------YRVAFLTSPAPNRRAIGDLRTVEEIGRVLRGRAAKVL
 Af1521    (86) MNLE--------------------------ER------GIKYVFHTVGPICSGM----WSEELKEKLYKAFLGPL

               852                                                                       861
hPARG26   (852) FLRPGVSSEN-----------------------------------------------------------------
 BtPARG   (853) FLRPGVSSEN-----------------------------------------------------------------
 RnPARG   (848) FLRPGVPPEN-----------------------------------------------------------------
 MmPARG   (845) FLRPGVPSEN-----------------------------------------------------------------
 AtPARG   (369) FLNCSKAWEHQNIFMDEGDNEIQLVRNGRDSGLLRTETTASHRTPLNDVEMNREKPANNLIRDFYVEGVDNEDHE
 TtPARG   (351) GFKQE---DQ-----------------------------------------------------------------
 TcPARG   (207) AAARHHG--------------------------------------------------------------------
 Af1521   (125) EKAEEMG--------------------------------------------------------------------

                    P-loop                                              Macrodomain
               862                                                                       927
hPARG26   (862) LSAVATGNWGCGAFGGDARLKALIQILAA------AAAERDVVYFTFG---DSELMRDIYSMHIFLTERKLTVGD
 BtPARG   (863) LSAVATGNWGCGAFGGDARLKALIQILAA------AVAERDVVYFTFG---DSELMRDIYSMHTFLTERKLTVGE
```

```
RnPARG   (858) LSAVATGNWGCGAFGGDARLKALIQLLAA------AAAERDVVYFTFG---DSELMRDIYSMHTFLTERKLNVGK
MmPARG   (855) LSAVATGNWGCGAFGGDARLKALIQILAA------AAAERDVVYFTFG---DSELMRDIYSMHTFLTERKLDVGK
AtPARG   (444) DDGVATGNWGCGVFGGDPELKATIQWLAAS-----QTRRPFISYYTFG----VEALRNLDQVTKWILSHKWTVGD
TtPARG   (358) LKTISTGKWGCGAFLGVFDLKFAIQWIAS------SRSNKKMIICTFQ---DEQTTKQIQQVFDLYK--QKNASI
TcPARG   (214) HRRLVLGAWGCGVFGNDPAQVAETFAGLLLDGGPFAGRFAHVVFAVWDTAPGAPRHAAFARRFGSL---------
Af1521   (132) VESIAFPAVSAGIYGCDLEKVVETFLEAVK--NFKGSAVKEVALVIYDRKSAEVALKVFERSL------------
```

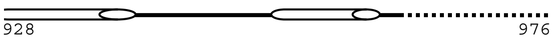

```
               928                                             976
hPARG26  (928) VYKLLLRYYNEECRNCSTPGPDIKLYPFIYHAVESCAETADHSGQRTGT
BtPARG   (929) VYKLLLRYYNEECRNCSTPGPDIKLYPFIYHAVESCTQTTNQPGQRTGA
RnPARG   (924) VYRLLLRYYREECRDCSSPGPDTKLYPFIYHAAESSAETSDQPGQRTGT
MmPARG   (921) VYKLLLRYYNEECRNCSTPGPDIKLYPFIYHAVESSAET----------
AtPARG   (510) LWNMMLEYSAQRLYKQTSVGFFSWLLPSLATTNKAIQPP----------
TtPARG   (422) FLKLVMDYPNSKY----MEDYTLLEYLIELGKEKATSKNS---------
TcPARG   (279) -------------------------------------------------
Af1521   (192) -------------------------------------------------
```
