# Supplementary figures and images for: Structures of the Human Poly (ADP-Ribose) Glycohydrolase Catalytic Domain Confirm Catalytic Mechanism and Explain Inhibition by ADP-HPD Derivatives
Source: PLoS One. 2012 Dec 10;7(12):e50889. doi: 10.1371/journal.pone.0050889 (PMC3519477; doi:10.1371/journal.pone.0050889)

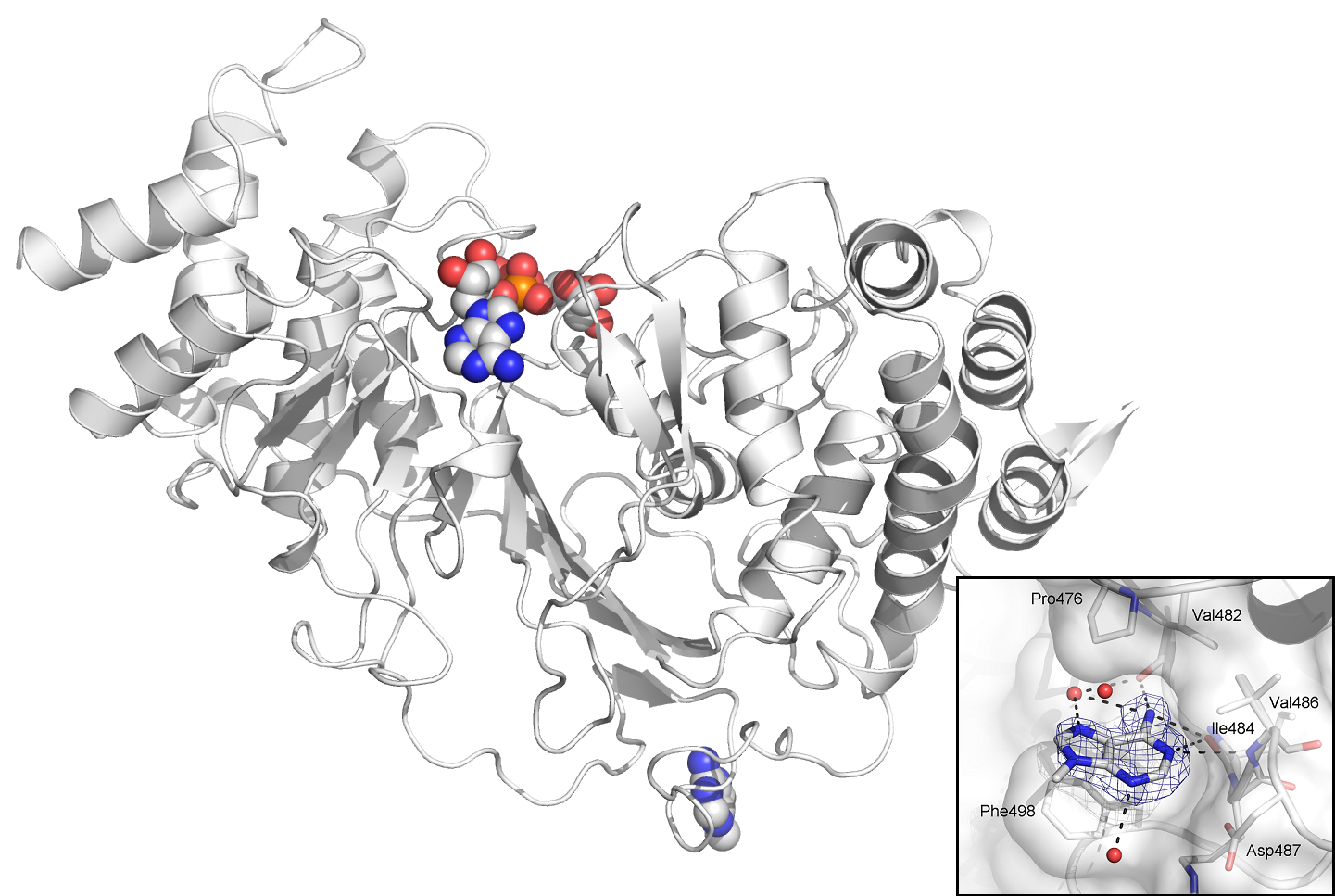

Supplement: Figure S3 — A second adenine binding pocket lies on the opposite face to the ADPR binding cleft. Bound ADPR and Adenine fragment are shown in spheres with carbon atoms in grey. Inset shows details of the secondary adenine binding site with adenine and selected PARG residues in stick representation (carbons in grey). Final 2Fo-Fc electron density for bound adenine is shown contoured at 1σ. (TIF) [file pone.0050889.s003.tif]
