# Supplementary material for: Structures of the Human Poly (ADP-Ribose) Glycohydrolase Catalytic Domain Confirm Catalytic Mechanism and Explain Inhibition by ADP-HPD Derivatives
Source: PLoS One. 2012 Dec 10;7(12):e50889. doi: 10.1371/journal.pone.0050889 (PMC3519477; doi:10.1371/journal.pone.0050889)
Supplement: Table S1 — Mapping PARG mutational data onto the hPARG26 structure. (DOCX) [file pone.0050889.s004.docx]

**Supporting information for Tucker *et al*.; Structures of the human poly (ADP-ribose) glycohydrolase catalytic domain confirm catalytic mechanism and explain inhibition by ADP-HPD derivatives**

**Table S1. Mapping PARG mutational data onto the hPARG26 structure.**

At=*Arabidopsis thaliana*, Bt=*Bos taurus*, Hs=*Homo sapiens*, Tc = *Thermonospora curvata*

| **Mutation** | | | | **Equivalent residue in hPARG** | **Location and contacts** (see also Figure 7) | **Rationale** | **Reference** |
| --- | --- | --- | --- | --- | --- | --- | --- |
| **Species** | **Wild-type** | **Mutant** | **Effect** |  |  |  |  |
| Hs | Arg462 | Ala | 48% wild-type activity | Arg462 | N-terminal extension (MTS). Donates H-bond to backbone CO of Leu862; multiple water mediated contacts. | Part of extended N-terminal structure which packs across and buttresses adenine binding site. Potential destabilisation of α10-β13 loop. | [1] |
| Hs | Arg463 | Ala | 105% wild-type activity | Arg463 | N-terminal extension (MTS). Partially solvent exposed; water mediated contacts to Ile 458 & Asn461; donates H-bond to backbone CO of Gln731. | Unclear why limited effect compared to Arg462Ala. | [1] |
| Hs | Δ(1-M464) | n/a | 31% wild-type activity | Δ(1-M464) | N-terminal extension (β1-MTS). | Loss of extended structure which stabilises C-terminal extension and adenine pocket. | [1] |
| Hs | Arg466 | Ala | 248% wild-type activity | Arg466 | N-terminal extension (MTS). Surface exposed | Surface residue with well defined side-chain density. Unclear why mutation should enhance activity | [1] |
| Hs | Δ(1-G468) | n/a | 8.4% wild-type activity | Δ(1-G468) | N-terminal extension (β1-MTS). | Loss of N-terminal “anchor” to extended loop region, leading to destabilisation of C-terminal extension, adenine pocket and possibly also “Tyr-clasp”. | [1] |
| Hs | Arg470 | Ala | 144% wild-type activity | Arg470 | N-terminal extension (MTS). Surface exposed | Surface residue with poorly defined side-chain density. Unclear why mutation should enhance activity; possibly related to changes in surface charge. | [1] |
| Hs | Arg463 + Arg464 + Arg466 + Arg470 | Ala | 113% wild-type activity | Arg463 + Arg464 + Arg466 + Arg470 | N-terminal extension (MTS). See above | Sum of individual effects. Influence of surface charge? | [1] |
| Hs | Leu471 | Asp | 148% wild-type activity | Leu471 | N-terminal extension (MTS); packs against one face of Trp814 | Unclear why this mutation should enhance activity. Speculate loop rearrangement to accommodate. | [1] |
| Hs | Δ(1-P472) | n/a | <0.1% wild-type activity | Δ(1-P472) | N-terminal extension (β1-MTS) | Loss of conserved Leu471 and Pro472 which respectively pack against & accept an H-bond from Trp814 at base of “Tyr-clasp”; destabilisation effects then transmitted to Tyr795 in active site. | [1] |
| Hs | Leu471 + Leu473 | Asp | 4% wild-type activity | Leu471 + Leu473 | N-terminal extension (MTS); Leu473 solvent exposed | Unclear why this combination should abolish activity, when Leu471Asp alone has positive effects. | [1] |
| Hs | Leu471 + Leu473 + Leu474 | Asp | <0.1% wild-type activity | Leu471 + Leu473 + Leu474 | N-terminal extension (MTS); Leu471 & Leu474 pack against faces of Trp814 | Leu474 in hydrophobic environment (Val478, Ile484, Val486, Leu690); substitution with Asp likely to disrupt local folding; effect transmitted to catalytic site via Leu471-Trp814-Leu474 contacts at base of “Tyr-clasp” | [1] |
| Hs | Δ(1-P477) | n/a | <0.1% wild-type activity | Δ(1-P477) | N-terminal extension (β1-MTS) | Loss of conserved Leu471, Pro472 & Leu474 which shield Trp814 at base of “Tyr-clasp” from solvent. Destabilisation of N-terminal extension propagates to Tyr795 in active site. | [1] |
| Hs | Lys616 + Gln617 + Lys618 + Glu688 + Lys689 + Lys690 | Ala | No effect | Lys616 + Gln617 + Lys618 + | β turn linking α5 to β6 | Surface loop; side-chains are well ordered in rPARG and mPARG structures with Lys618 involved in crystal contacts; Lys616 & Gln617 donate H-bonds to Asn508 & Val774 backbone CO respectively | This work |
|  |  |  |  | Glu688 + Lys689 + Lys690 | C-terminus of α5 & α6-β6 loop | Surface exposed; side-chains are well-defined in unliganded rPARG structure despite limited contacts; greater degree of disorder in the rPARG-ADP-HPD & mPARG structures with relatively high B-factors & poorly-defined density |  |
| Bt | Glu708 | Asn | No effect on catalytic activity; 2.5-fold increase in affinity for 8-AH-ADP-HPD | Glu707 | Surface-exposed (β6-β7 loop) | Surface-exposed; distant from catalytic site. Unclear from structure why affinity for 8-AH-ADP-HPD enhanced. | [2] |
| Bt | Glu728 | Asn | 18% wild-type specific activity;  2.5-fold increase in affinity for 8-AH-ADP-HPD | Glu727 | Buried; accepts H-bonds from adenine 6-NH_2_ group and Tyr792-OH | Impact on H-bond network to adenine, Tyr792 and, via waters, residues in diphosphate binding site; increased affinity for 8-AH-ADP-HPD binding may result from increased flexibility in the “Tyr-clasp” due to alterations in H-bond network to Tyr792. | [2] |
| Bt | Asp738 | Asn | No detectable activity; 1.4-fold decrease in affinity for 8-AH-ADP-HPD | Asp737 | Buried; donates H-bond to Oε2 and contacts Cγ of Glu755 in conserved QEE motif | Minor alteration in the Glu755 side-chain conformation. Potential impact on the proton-relay system proposed by Dunstan and co-workers [3]. | [2] |
| Hs | Asn740 | Ala | Specific activity ~30% of wild-type | Asn740 | Donates H-bond to ribose” 3”-OH; accepts H-bond from backbone NH of Phe742. | Destabilisation of ribose” orientation, conformation and binding through loss of H-bond to 3”-OH. | [4] |
| At | Gly262 | Glu | Increased accumulation of PAR chains | Gly744 | First Gly of conserved GGG motif in catalytic centre; buried by Phe759 side-chain; donates H-bond to Glu755 Oε2. | Backbone phi/psi incompatible with non-Gly residue; no space to accommodate side-chain; leading to disruption of ribose” binding site through destabilisation of GGG loop and consequent destabilisation of Glu755 & Glu756. | [5] |
| Bt | Glu756 | Asn | No detectable activity; 8-fold increase in 8-AH-ADP-HPD affinity | Glu755 | Accepts H-bond from ribose” 2”-OH, Asn737 side-chain NH_2_ and backbone NH of Asn740; contacts GGG motif | Effects on H-bond network to Asn737, Asn740 and Gly744-Gly745 likely to disrupt proposed “proton-relay” network [4] linking Asp737-Glu755-Glu756. Mutation to Asn has limited effect on shape of HPD pocket & may retain H-bond to C4-OH, hence binding of 8-AH-ADP-HPD maintained, however, unclear why affinity should increase. | [2] |
| Hs | Glu755 | Ala | No detectable activity |  |  | Loss of H-bonds to ribose” 2”-OH, Asn737 & Asn740 disrupts stabilisation and orientation of ribose” conformation and proton relay network. | [4] |
| Tc | Glu114 | Ala | No detectable activity; 10-fold decrease in ADPR affinity |  |  |  |  |
| Bt | Glu757 | Asn | No detectable activity; no effect on 8-AH-ADP-HPD affinity | Glu756 | Accepts H-bond from ribose” 1”-OH and backbone NH of Gly746 & Val753 | Proposed catalytic acid/base; protonates leaving group and activates bound waters for nucleophilic attack.  Lack of effect on 8-AH-ADP-HPD binding due to absence of 1”-OH (and thus lack of interactions). Unclear why affinity for ADPR unaffected. | [2] |
| Hs | Glu756 | Ala | No detectable activity |  |  |  | [4] |
| Tc | Glu115 | Ala | No detectable activity; no effect on ADPR affinity |  |  |  |  |
| Bt | Glu765 | Asn | Specific activity 29% of wild-type | Glu764 | Buried in hydrophobic core (α8-α9 loop); accepts H-bonds from Glu787 backbone NH, Gln627-NH2, Tyr789-OH and buried water molecules | Destabilisation of overall fold | [2] |
| Bt | Glu774 | Asn | Specific activity 61% of wild-type | Glu773 | Partially solvent exposed (α9-β9 loop); accepts H-bonds from Gln617 backbone NH (α4-β5 loop) & Ser655-OH (α5-α6 loop) | Destabilisation of overall fold | [2] |
| Bt | Glu780 | Asn | Specific activity 57% of wild-type | Glu779 | Buried (β9); salt-bridge to Arg699; accepts H-bonds from backbone NH of Lys618 & Asp776 | Destabilisation of overall fold | [2] |
| Bt | Glu788 | Asn | None | Glu787 | Partially surface-exposed (β9-β10 loop); accepts H-bonds from Gln627-NH2 & three water molecules; contacts Arg817; stacks against Tyr789 | Partially surface-exposed; minimal disruption to fold; distant from active site although potential for transmission of effects as located within “Tyr-clasp”. | [2] |
| Bt | Tyr796 | Ala | 8-fold reduction in catalytic activity; 20-fold reduction in 8-AH-ADP-HPD affinity | Tyr795 | Apex of “Tyr-clasp” (β10-β11 loop); forms one face of the adenine pocket; displaced by 8-OA substituent of OA-ADP-HPD | Network of direct and water-mediated interactions from Tyr795-OH to ADPR α-phosphate & Gln754 backbone NH; disruption of H-bond network destabilises β-turn containing Tyr795 (“Tyr-clasp”), α-phosphate binding & QEE motif; loss of Tyr side-chain increases solvent accessibility of adenine site. | [6] |
|  |  | Trp | Limited effect on catalytic activity and 8-AH-ADP-HPD binding |  |  | Bulkier Trp could be accommodated by limited rearrangement, however, indole NH unlikely to completely mimic interactions of Tyr hydroxyl, suggesting role of Tyr795 in closing one face of adenine binding pocket and stacking against adenine may be more important to catalysis than hydrogen bond network. | [6] |
| Hs | Ala874 | Trp | <50% wild-type activity | Ala874 | Tip of phosphate binding loop; donates H-bond from backbone NH to α-phosphate O | Destabilisation of phosphate binding loop and disruption of ribose” site combined with steric block of PAR attachment site | [4] |
| Hs | Phe875 | Ala | Catalytic activity abolished | Phe875 | Tip of phosphate binding loop; forms one face of ribose” binding site; donates H-bond from backbone NH to α-phosphate O | Disruption of ribose” and phosphate binding sites | [4] |

**Supplemental References.**

1. Botta D, Jacobson MK. (2010) Identification of a regulatory segment of poly(ADP-ribose) glycohydrolase. Biochemistry (N Y ) 49(35): 7674-7682.

2. Patel CN, Koh DW, Jacobson MK, Oliveira MA. (2005) Identification of three critical acidic residues of poly(ADP-ribose) glycohydrolase involved in catalysis: Determining the PARG catalytic domain. Biochem J 388: 2-500.

3. Dunstan MS, Barkauskaite E, Lafite P, Knezevic CE, Brassington A, et al. (2012) Structure and mechanism of a canonical poly(ADP-ribose) glycohydrolase. Nat Commun 3: 878.

4. Slade D, Dunstan MS, Barkauskaite E, Weston R, Lafite P, et al. (2011) The structure and catalytic mechanism of a poly(ADP-ribose) glycohydrolase. Nature 477(7366): 616-620. <http://dx.doi.org/10.1038/nature10404>. Available: <http://ovidsp.ovid.com/ovidweb.cgi?T=JS&CSC=Y&NEWS=N&PAGE=fulltext&D=prem&AN=21892188> via the Internet.

5. Panda S, Poirier GG, Kay SA. (2002) Tej defines a role for poly(ADP-ribosyl)ation in establishing period length of the arabidopsis circadian oscillator. Dev Cell 3(1): 51-61.

6. Koh DW, Patel CN, Ramsinghani S, Slama JT, Oliveira MA, et al. (2003) Identification of an inhibitor binding site of poly(ADP-ribose) glycohydrolase. Biochemistry 42(17): 4855-4863.
